# Human SKI component SKIV2L regulates telomeric DNA-RNA hybrids and prevents telomere fragility

**DOI:** 10.1016/j.isci.2024.111096

**Published:** 2024-10-04

**Authors:** Emilia Herrera-Moyano, Rosa Maria Porreca, Lepakshi Ranjha, Eleni Skourti, Roser Gonzalez-Franco, Emmanouil Stylianakis, Ying Sun, Ruihan Li, Almutasem Saleh, Alex Montoya, Holger Kramer, Jean-Baptiste Vannier

**Affiliations:** 1 Telomere Replication & Stability Group, Institute of Clinical Sciences, Imperial College London, London W12 0NN, UK; 2 MRC London Institute of Medical Sciences, Du Cane Road, London W12 0NN, UK; 3 Biological Mass Spectrometry & Proteomics, MRC-LMS, Hammersmith Hospital Campus, London W12 0NN, UK; 4 DNA Replication Group, Institute of Clinical Sciences, Imperial College London, London W12 0NN, UK

**Keywords:** Molecular biology, Cell biology

## Abstract

Super killer (SKI) complex is a well-known cytoplasmic 3′–5′ mRNA decay complex that functions with the exosome to degrade excessive and aberrant mRNAs, is implicated with the extraction of mRNA at stalled ribosomes, tackling aberrant translation. Here, we show that SKIV2L and TTC37 of the hSKI complex are present within the nucleus, localize on chromatin and at some telomeres during the G2 cell cycle phase. In cells, SKIV2L prevents telomere replication stress, independently of its helicase domain, and increases the stability of telomere DNA-RNA hybrids in G2. We further demonstrate that purified hSKI complex binds telomeric DNA and RNA substrates *in vitro* and SKIV2L association with telomeres is dependent on DNA-RNA hybrids. Taken together, our results provide a nuclear function for SKIV2L of the hSKI complex in overcoming replication stress at telomeres mediated by its recruitment to DNA-RNA hybrid structures in G2 and thus maintaining telomere stability.

## Highlights

• The human SKI complex, involved in mRNA decay, is recruited to telomeres in G2
• SKIV2L plays a protective role in telomere maintenance
• The recruitment of SKIV2L to telomeres is dependent on DNA-RNA hybrids
• SKIV2L facilitates telomeric DNA-RNA hybrid regulation in G2

## Graphical abstract

**Figure:**
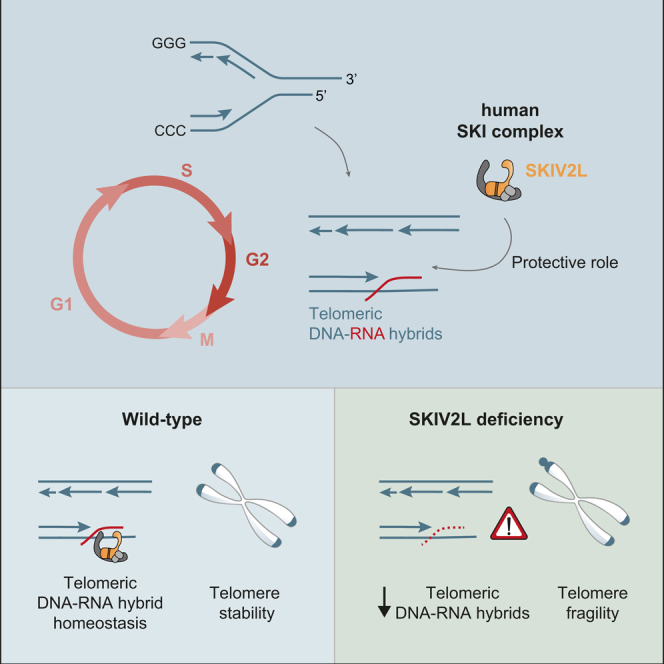

## Introduction

To tackle the challenge of aberrant or excessive cytoplasmic and nuclear RNA molecules, cells have evolved different RNA decay pathways. Among these, the nonsense-mediated mRNA decay (NMD) surveillance pathway is involved in the degradation of mRNAs presenting premature translation termination^1^ and also works as a quality-control system regulating the expression of physiological RNAs.^2^ Although human NMD has been widely investigated for its cytoplasmic functions,^3^ some of its core components have been identified in the nucleus, targeting several essential biological processes including telomere homeostasis.^4^^,^^5^

Telomeres are transcribed by RNA polymerase II from subtelomeric regions toward the chromosome ends into the long non-coding telomeric repeat-containing RNA (TERRA).^5^^,^^6^^,^^7^ TERRA transcription and its association with telomeres is co-ordinated throughout the cell cycle to allow for correct post-replication processing of telomeres but also to avoid collisions between DNA replication and transcription.^8^^,^^9^ Due to the complementary nature of TERRA to telomeric DNA, R-loops, constituted by the DNA-RNA hybrid and the displaced DNA strand, may form at telomeres and result in telomeric DNA replication stress while DNA-RNA hybrids also act as key mediators of telomere length dynamics.^10^^,^^11^^,^^12^^,^^13^ In this sense, some factors such as the DNA helicase RTEL1 in mammalian cells or the RNA binding protein Npl3 in yeast have been described to promote TERRA DNA-RNA hybrid association with telomeres.^14^^,^^15^ TERRA levels vary in a cell cycle-dependent manner, peaking in G1 phase, decreasing in S phase and reaching the lowest level in S/G2 in HeLa cells,^8^ which suggests the presence of a regulatory mechanism. Indeed, the RNA helicase UPF1, essential for the NMD pathway, binds to chromatin during S phase^4^ and particularly to telomeres *in vivo.*^5^ Its activity is essential to ensure replication and telomere length homeostasis by mediating the displacement of TERRA from telomeres.^16^

In *Saccharomyces cerevisiae*, the cytoplasmic 3′–5′ mRNA decay relies on the super killer (SKI) and exosome complexes. The yeast SKI tetramer is composed of Ski2, Ski3 and two subunits of Ski8 (SKIV2L, TTC37, WDR61 in human, respectively).^17^ Ski2 presents a 3′ to 5′ RNA helicase activity that channels the RNA into the exosome for further degradation via interaction with Ski7.^17^^,^^18^ Consistent with the function of its yeast homolog, human SKIV2L facilitates cytoplasmic degradation of mRNAs and viral RNA by the exosome and plays a role in RNA interference.^19^^,^^20^^,^^21^^,^^22^ Recently, the human SKI (hSKI) complex was shown to extract mRNA by directly interacting with the ribosomal complexes *in vitro*.^23^ Indeed, SKIV2L works in a translation surveillance program with the RNA binding protein AVEN, preventing ribosome stalling by eliminating RNA transcripts.^24^ The implication of hSKI in different RNA regulatory pathways and its localization in both the cytoplasm and the nucleus,^25^^,^^26^ suggests a possible role for the hSKI complex in nuclear RNA surveillance.

Our data show that the hSKI complex also localizes to chromatin and is enriched at some telomeres during the G2 phase of the cell cycle. Purified hSKI not only interacts with RNA but also has a high affinity for single-stranded telomeric DNA. Strikingly, hSKI binds DNA-RNA hybrids *in vitro* and its recruitment to telomeres is dependent on telomeric DNA-RNA hybrids. At difficult-to-replicate telomeres, SKIV2L promotes the stabilization or protection of DNA-RNA hybrids that favor telomere biology independently on its helicase activity, while its loss decreases the amount of those structures in G2 and provokes telomere fragility. Overall, our results describe an unforeseen function of SKIV2L in the maintenance of telomere integrity before mitosis by overcoming replication stress through the regulation of telomeric DNA-RNA hybrids homeostasis.

## Results

### The human SKI (hSKI) complex is recruited to telomeres, particularly in G2

**Figure 1:**
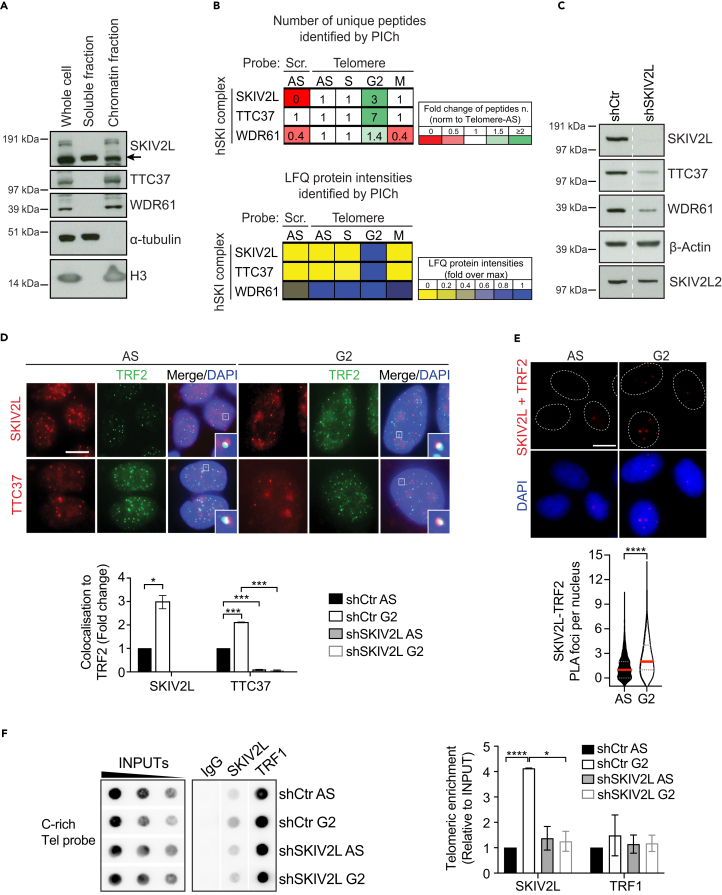
SKIV2L of the hSKI complex is present at telomeres in G2 (A) Subcellular fractionation assay in asynchronous HeLa1.3 cells. (B) Proteomics of isolated chromatin segments analysis showing the binding of hSKI to telomeres throughout the cell cycle. Tables are listing the number of unique peptide numbers isolated, including the fold change values of unique peptides normalized to the asynchronous values (top panel) and the relative LFQ intensity values identified by PICh. AS: asynchronous. Scr: scramble. (C) WB of hSKI (SKIV2L, TTC37, and WDR61) and SKIV2L2 in shCtr (Control) or shSKIV2L HeLa1.3 cells. (D) Immunofluorescence of pre-extracted cells showing co-localization of SKIV2L and TTC37 with TRF2 in asynchronous (AS) and G2-synchronized cells. % of total number of SKIV2L or TTC37 foci colocalizing with TRF2 are normalized to shCtr AS samples (means ± SEM, *n* = 2 independent experiments, scale bar 15 μm). t test ∗*p* < 0.05, ∗∗∗*p* < 0.001. (E) Proximity ligation assay of SKIV2L-TRF2, showing increasing number of foci in G2 synchronized cells (median, Q1 and Q3, 707 (AS) and 638 (G2) cells scored per condition, 3–4 independent experiments, scale bar 10 μm). Mann-Whitney U test ∗∗∗∗*p* < 0.0001. (F) ChIP-dot blot of SKIV2L and TRF1 in AS and G2-synchronized shCtr and shSKIV2L cells (means ± SEM, *n* = 3). t test ∗*p* < 0.05, ∗∗∗∗*p* < 0.0001. See also Figure S1.

In order to assess the localization of hSKI within HeLa long telomeres (HeLa1.3) cells, we firstly employed cell fractionation. The three components of hSKI: SKIV2L, TTC37, and WDR61 were detected in the whole cell lysate and the chromatin-bound fraction, while the soluble fraction only contained SKIV2L (Figure 1A). Because other factors of the NMD pathway have been associated with telomeres in S phase,^16^ we assessed the binding of hSKI components to telomeres throughout the cell cycle. Upon cell synchronization (Figures S1A and S1B), telomere binding was examined using proteomic of isolated chromatin segments (PICh).^27^^,^^28^ The PICh analysis was conducted using unique peptides identified in each condition and the respective label-free quantification values. As expected, peptides from the Shelterin factors: TRF2, TRF1, RAP1, TIN2, TPP1 and POT1 were found enriched in the reads obtained with the telomeric probe compared to the scrambled probe used as a control, which confirms specificity of the experiment (Figure S1C). Interestingly, the three components of the hSKI complex were also found in the telomere probe-derived peptide reads, with SKIV2L and TTC37 significantly enriched during G2 phase (Figure 1B) while WDR61 seems distributed evenly across the cell cycle, which could be related to its involvement within the PAF complex.^29^

To further validate the presence of the hSKI complex at telomeres, we successfully generated stable SKIV2L knockdown (shSKIV2L) and control (shCtr) HeLa1.3 cells using short hairpin RNAs (Figure 1C). Notably, the depletion of SKIV2L was accompanied by decreased protein levels of the other two hSKI components (TTC37 and WDR61) but had no effect on the protein expression of its paralog, SKIV2L2, known to regulate telomerase RNA levels.^30^ Using immunofluorescence, we examined the co-localization of the two exclusive components of hSKI, SKIV2L, and TTC37, with the telomeric protein TRF2. SKIV2L and TTC37 were detected in the nucleus, and at a fraction of telomeres in G2 cells while barely detectable at telomeres in asynchronous cells, consistently with the PICh data (Figure 1D). In agreement with the reduced protein levels upon SKIV2L depletion (Figure 1C), we observed loss of IF signals for SKIV2L and TTC37, while TRF2 remained unchanged (Figures 1D, S1D, S1E, and S2A). Similarly, we observed localization of SKIV2L to some telomeres using SKIV2L-immunofluorescence coupled with telomeric FISH in G2-synchronized cells, which is abolished upon SKIV2L depletion (Figure S1F). In addition, we confirmed the enhanced SKIV2L-TRF2 co-localization in G2 phase by proximity ligation assay (PLA) (Figures 1E and S1G). PLA with individual antibody (TRF2 or SKIV2L) and together in the SKIV2L knockdown cell line was used to ensure specificity of the results (Figure S1G). Finally, telomeric ChIP-dot blot confirmed the enriched presence of SKIV2L at some telomeres in G2 cells compared to almost nothing in AS, which is significantly reduced in SKIV2L-depleted G2 cells (Figure 1F). Notably, we did not notice any alteration in the recruitment of TRF1 and TRF2 to telomeres, respectively, by ChIP and IF (Figures 1F and S2A), and no changes in TRF1, TRF2, and TIN2 protein expression by western blot (Figure S2B), in cells deficient for SKIV2L. This suggests that SKIV2L downregulation has no major effect on the expression of shelterin proteins.

Taken together, these results indicate that SKIV2L localizes to some telomeres particularly in G2 phase of the cell cycle in HeLa1.3 cells.

### SKIV2L suppresses telomere fragility and DNA damage

**Figure 2:**
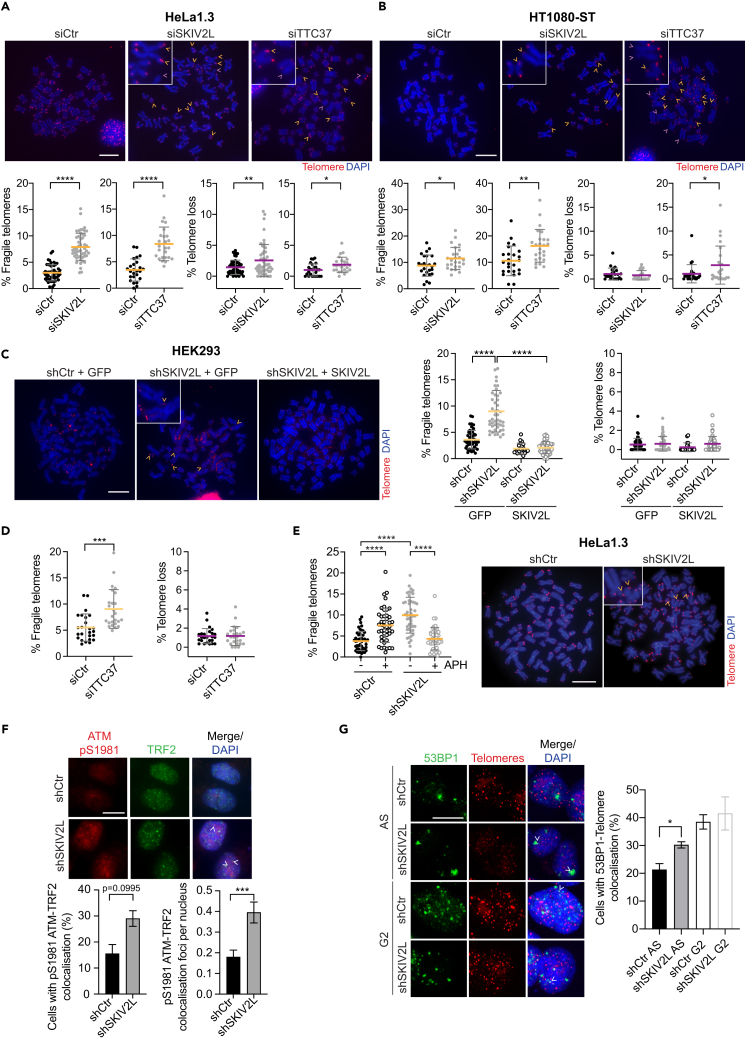
SKIV2L and TTC37 prevents telomere fragility (A and B) Telomere FISH analysis in SKIV2L- and TTC37-depleted HeLa1.3 and HT1080-ST cells using siRNAs: % of telomere fragility (yellow) and loss (purple) per metaphase in siCtr, siSKIV2L and siTTC37 (means ± SD, *n* > 25 metaphases, 2 independent experiments, scale bar, 10 μm). t test ∗*p* < 0.05, ∗∗*p* < 0.01, ∗∗∗∗*p* < 0.0001. (C) Telomere FISH analysis in shCtr (Control) or shSKIV2L HEK293 cells expressing GFP or SKIV2L (means ± SD, *n* > 25 metaphases, 2 independent experiments, scale bar, 10 μm). Details are as in (A). (D) Telomere FISH analysis in TTC37-depleted HEK293 cells using siRNAs (means ± SD, *n* = 25 metaphases). Details are as in (A). (E) Telomere FISH analysis in HeLa1.3 cells using shRNAs: shCtr and shSKIV2L treated with DMSO (−, control) or APH (+) (*n* > 45 metaphases, 2 independent experiments). Details are as in (A). (F) IF of pre-extracted cells showing co-localization of pS1981 ATM autophosphorylation with TRF2 (telomeres) in shCtr and SKIV2L-depleted (shSKIV2L) HeLa1.3 cells. Quantification of the percentage of cells with co-localization foci and mean number of foci per nucleus is depicted (means ± SEM, *n* = 200 cells, 2 independent experiments, scale bar 15 μm). t test ∗∗∗*p* < 0.001. (G) IF-FISH of pre-extracted cells showing co-localization of 53BP1 with telomeres in asynchronous (AS) or G2-synchronized control (shCtr) and SKIV2L-depleted (shSKIV2L) HeLa1.3 cells. Quantification of the percentage of cells with co-localization foci is depicted (means ± SEM, *n* > 400 cells, 3 independent experiments, scale bar 15 μm). t test ∗*p* < 0.05. See also Figure S2.

To shed light on the potential telomeric function of hSKI, we investigated whether the depletion of the hSKI components would perturb telomere stability. We used telomere quantitative-FISH analysis of metaphases of HeLa1.3, HT1080-ST, and HEK293 cells depleted for either SKIV2L, TTC37, or WDR61 by siRNA or shRNAs to look for telomere abnormalities (Figures 2A–2D and S2C–S2F). We noticed an increase in telomere fragility, marker of telomere replication stress,^31^^,^^32^ in all three tested cell lines depleted for SKIV2L and TTC37. However, the effect of SKIV2L and TTC37 knock-downs on telomere loss was not consistent across the three cell lines and therefore was not followed up. Notably, the depletion of the third component of the complex, WDR61, did not induce significant telomere fragility in HeLa1.3 or HT1080-ST cell lines (Figures S2C and S2F) consistent with its differential association with telomeres throughout the cell cycle and its dual role as part of the hSKI and PAF complexes.^26^ In order to validate the direct effect of SKIV2L loss on telomere fragility we complemented HEK293 shSKIV2L stable cell line with WT SKIV2L (shRNA resistant; Figures 2C and S2D). The expression of SKIV2L in HEK293 did not have an effect on telomere phenotypes and the complementation restored telomere fragility to basal levels, ensuring specificity of the targets and effects. The same induction of telomere fragility was reproduced in the stable shSKIV2L HeLa1.3 cell line showing more than 2.5-fold increase compared to the shCtr (Figure 2E). To test whether this phenotype is dependent on replication stress at telomeres, we treated HeLa1.3 stable cell lines with low doses of the DNA polymerase inhibitor aphidicolin (APH). Telomere fragility caused by SKIV2L depletion is slightly more pronounced than in control cells treated with APH (used as a positive control), which supports the idea that SKIV2L is important for the maintenance of telomere integrity. Surprisingly, SKIV2L-depleted cells treated with APH showed suppression of telomere fragility to background levels (Figure 2E). This reduction in the frequency of APH-induced fragile telomeres was previously reported following depletion of the mouse CTC1 component of the CST complex, which facilitates telomere replication.^33^ Recent evidence suggests that telomere fragility is not caused by the primary disruption of DNA replication, but it arises as a consequence of the repair of truncated replication forks by the homologous recombination machinery.^34^^,^^35^ Our data suggest that telomere DNA replication is still disrupted in SKIV2L-depleted cells treated with APH, as evidenced by the observation of the telomere loss phenotype (Figure S2G). However, the processing of the associated DNA replication intermediates may not lead to the expression of the fragile telomere phenotype under these conditions.

Given that all the tested cells lines in which SKIV2L proved to prevent telomere instability are positive for telomerase expression, we asked whether SKIV2L function would also be necessary in primary cells or in cells that rely on the alternative lengthening of telomeres (ALT) pathway. We observed an increase in telomere fragility upon SKIV2L depletion in primary IMR90 cells (Figures S2H and S2I) confirming that the telomeric role of SKIV2L is not restricted of telomerase-positive cells. On the contrary, SKIV2L depletion does not lead to any further effect on telomere fragility in ALT-positive U2OS cells (Figures S2J and S2K). This result suggests that the function of SKIV2L preserving telomere stability is dispensable in ALT cells, which have intrinsically elevated levels of replication stress, even higher than HeLa1.3 cells treated with APH.

Since we identified various telomere abnormalities in SKIV2L knockdown HeLa1.3 cells, we asked if this is accompanied with the activation of the DNA damage response. We observed an increase in the levels of activated ATM (pS1981 ATM autophosphorylation) at telomeres via co-localization with TRF2 upon SKIV2L depletion (Figure 2F). Furthermore, to measure the levels of DNA damage at telomeres, we quantified telomeric dysfunction-induced foci (TIFs) by the localization of 53BP1 foci at telomeres.^36^ In SKIV2L-depleted asynchronous cells, we observed a slightly increased levels of TIFs when compared to control cells (Figure 2G). However, this increase was not observed in G2-synchronized cells, suggesting that 53BP1 signaling upon SKIV2L depletion might correspond to 53BP1 nuclear bodies derived from replication stress during the previous cell cycle.^37^ To test whether the observed phenotypes are related to a general impact of SKIV2L in cell cycle progression we assessed cell cycle profile of HeLa1.3 cells depleted of SKIV2L, both in normal conditions and in the presence of low doses of aphidicolin. As expected, APH-treated cells exhibit a delay in the progression through the S phase, while SKIV2L knockdown has not a further effect on the cell cycle profile of HeLa1.3 cells in any of the tested conditions (Figure S2L). In summary the aforementioned results confirm that SKIV2L is a regulator of telomere maintenance and its absence leads to abnormal replication of telomeres in telomerase-positive and primary cells.

### SKIV2L ATP binding and helicase motifs are dispensable for its function at telomeres

**Figure 3:**
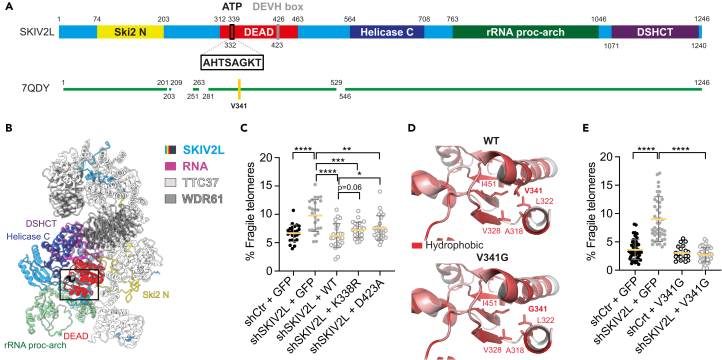
SKIV2L helicase activity is dispensable for the suppression of telomere fragility (A) Schematic diagram to illustrate the domain organization and features of human SKIV2L. The structurally resolved (via cryo-EM) regions of the SKIV2L subunit extracted from the RNA-bound human SKI complex in the closed state (PDB: 7QDY) is represented by a green colored bar. The SKIV2L subunit harbors the following domains: Ski2 N-terminal (Ski2 N) domain, DEAD/DEAH box helicase (DEAH) domain, ATP binding (ATP) domain, Helicase C-terminal (Helicase C) domain, rRNA-processing arch (rRNA proc-arch) domain and DOB1/SK12/helY-like DEAD box helicases C-terminal (DSHCT) domain. The SKIV2L-V341 residue is highlighted in yellow. (B) Overview of the structure of the RNA-bound human SKI complex in the closed state, featuring: the helicase SKIV2L, tetratricopeptide repeat protein 37 (TTC37) and WD repeat-containing protein 61 (WDR61). (C) Telomeric FISH analysis: % of telomere fragility in shCtr and shSKIV2L HEK293 cells expressing GFP or SKIV2L (WT), SKIV2L-K338R, SKIV2L-D423A (means ± SD, *n* > 20 metaphases, 2 independent experiments). t test ∗*p* < 0.05, ∗∗*p* < 0.01, ∗∗∗*p* < 0.001, ∗∗∗∗*p* < 0.0001, significance is not shown for comparisons with *p* > 0.06. (D) Comparison of the structure of the wild-type (WT) SKIV2L DEAD domain and a mutant structure, generated using Missense3D, featuring a valine to glycine 341 mutant. In the WT structure, V341 forms hydrophobic contacts with: A318, L322, V328 and I451. The hydrophobic surface shown is colored according to the Eisenberg hydrophobicity scale. (E) Telomeric FISH analysis: % of telomere fragility in shCtr and shSKIV2L HEK293 cells expressing GFP or SKIV2L-V341G (means ± SD, *n* > 20 metaphases, 2 independent experiments). t test ∗∗∗∗*p* < 0.0001. See also Figure S3.

The SKIV2L subunit of the hSKI complex belongs to the Ski-2 like family of RNA helicases and contains a conserved ATPase domain (Figures 3A and 3B). Given that the SKI complex is conserved in evolution and yeast Ski can unwind RNA duplexes in 3′ to 5′ direction,^17^ we assessed the potential of this region for ATP binding by comparing the primary sequence and 3D structural organization of human SKIV2L with that of the yeast homolog Ski2 (Figure S3A).^38^ We next assessed the requirement of the ATP binding and DEVH box helicase motifs for SKIV2L function at telomeres by complementing SKIV2L-deficient HEK293 stable cells (shSKIV2L) with GFP, WT or SKIV2L-K338R, and -D423A. SKIV2L-K338R and -D423A point mutants are expected to affect ATP release and helicase activity, as they affect key residues of the ATP binding and DEVH box helicase motifs, respectively^39^^,^^40^ (Figures 3A, 3B, and S3A). We observed a suppression of the telomere fragility phenotype with the expression of SKIV2L-K338R and, to a lesser extent, -D423A mutants in shSKIV2L cells to the same basal levels of control cells (Figures 3C and S3B). SKIV2L point mutant V341G is localized only 3 amino acid residues downstream of the SF2 helicase ATP binding site (Figure 3A) and identified as a missense mutation in the rare autosomal recessive disorder trichohepatoenteric syndrome (THES).^41^^,^^42^ The valine residue (V341) resides on an alpha helix which is involved in ATP binding (Figures 3D and S3A) and is neighboring lysine K338. Then, using the structure of the human SKIV2L protein, we predicted, via the Missense3D mutant predication server,^43^ that the valine to glycine substitution will provoke significant damaging structural changes (Figure 3D). The substitution is predicted to provoke a change between the buried and exposed state of the target variant residue. The valine residue is buried with 0.0% relative accessible surface area while the glycine substitution exposes the residue to 17.8%. The wild-type valine residue also forms important hydrophobic contacts with several residues: A318, L322, V328, and I451. The mutation disrupts this interaction and is predicted to impact the binding of ATP in this region. Overall, the structural analysis of V341G substitution predicts weaker ATP binding. Similar to WT SKIV2L (Figure 3C), SKIV2L-V431G complemented the SKIV2L deficient cells and suppressed telomere fragility (Figure 3E). Together, this suggests that the action of SKIV2L to prevent telomere fragility is independent of its ATP binding and DEVH box helicase motifs.

### Purified hSKI complex binds RNA and DNA substrates containing telomeric repeats

**Figure 4:**
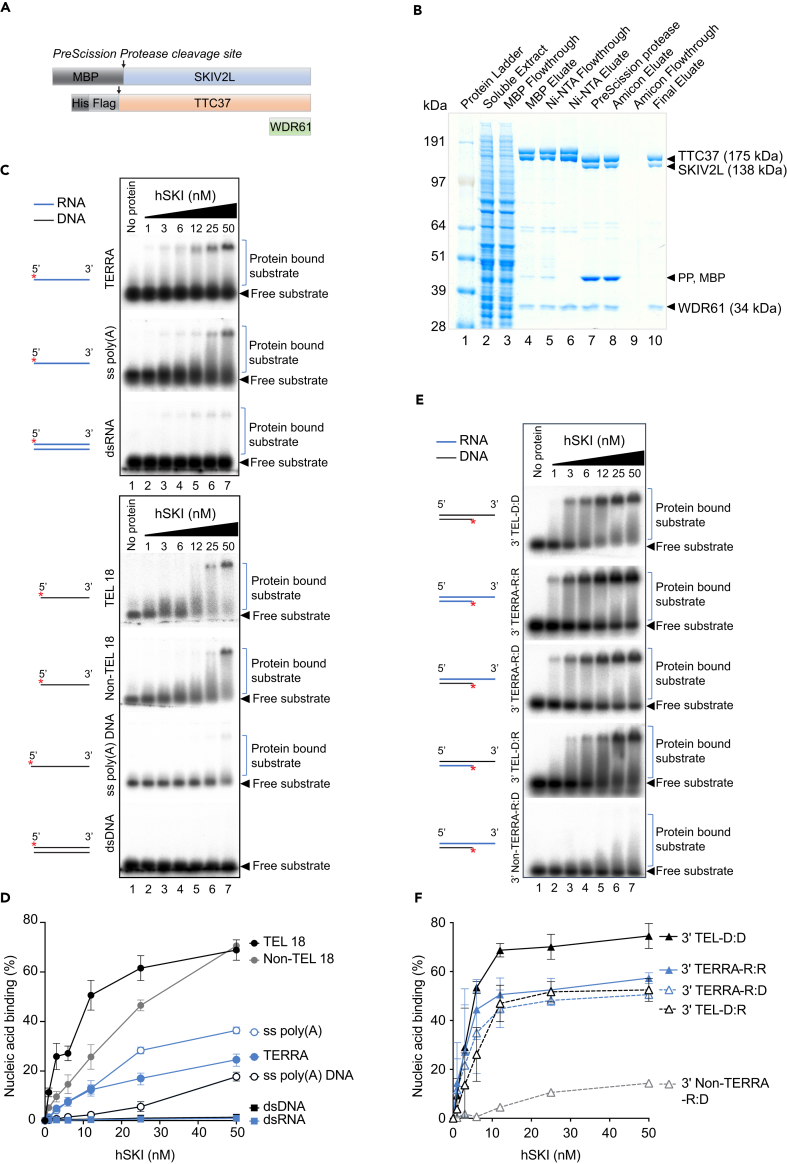
Purified recombinant hSKI binds preferentially ssDNA and telomeric DNA-RNA hybrids containing 3′ overhangs *in vitro* (A) hSKI constructs. MBP, Maltose binding protein; His, 6x histidine; Flag, 3x flag. (B) Coomassie blue SDS-PAGE gel showing purified hSKI (lane 10) and different purification fractions. PP, PreScission protease. (C) Electrophoretic mobility shift assays showing binding of hSKI to different RNA and DNA substrates. Blue lines denote RNA, black lines denote DNA and asterisk indicates radioactive ^32^P label. hSKI protein amounts are as indicated (nM) (D) Quantification of (C) showing % of nucleic acid binding calculated as protein bound substrate signal relative to free substrate signal (mean ± SEM, n = 2–4). (E) Electrophoretic mobility shift assays showing binding of hSKI complex to different 3′ and 5′ overhang RNA/DNA substrates. Blue lines denote RNA, black lines denote DNA and asterisk indicates radioactive ^32^P label. (F) Quantification of (E) showing % of nucleic acid binding calculated as protein bound substrate signal relative to free substrate signal (mean ± SEM, *n* = 2). See also Figure S4.

To elucidate the potential nuclear function of hSKI and understand how it interacts with telomeres, we purified the complex and carried out *in vitro* biochemical assays. We designed constructs featuring N-terminus MBP-tagged SKIV2L, N-terminus His-Flag tagged TTC37, untagged WDR61 and purified from insect cells the hSKI complex by subsequent affinity and size exclusion chromatography (Figures 4A and 4B).

As SKIV2L contains two putative RecA-like domains and interacts with mRNA, we set out to evaluate the binding properties of purified hSKI complex to various nucleic acids using electrophoretic mobility shift assays (Figure 4C). First, we found that similar to its yeast homolog, hSKI can bind single stranded RNA (ssRNA) containing a poly(A) tail but also ssDNA of similar sequence (ss poly(A) and ss poly(A) DNA, respectively) (Figures 4C and 4D). Since we observed that hSKI localizes to telomeres, we then evaluated the binding affinity of hSKI to various telomeric and non-telomeric RNA and DNA substrates including ssRNA and ssDNA (TERRA, TEL 18, and non-TEL 18) and double-stranded RNA and DNA (dsRNA/dsDNA) (Figure 4C). Strikingly, we found that hSKI favors binding to telomeric ssDNA (TEL18, K_d_ = 12 nM) as compared to non-telomeric ssDNA (Non-TEL18, K_d_ = 30 nM) of similar length (Figures 4C and 4D). Binding to ssRNA of telomeric sequence, that mimics TERRA, was also observed; however, the affinity was lower than for telomeric ssDNA (TERRA vs*.* TEL18) (Figures 4C and 4D). No binding to blunt-ended dsDNA and dsRNA was observed. These results show that hSKI can bind single-stranded telomeric oligonucleotides, suggesting that it may interact with telomeric structures *in vivo*.

### Purified hSKI binds telomeric DNA-RNA hybrids with single-stranded 3′ end

Previously, SKIV2L and TTC37 were identified among 400 factors enriched in a synthetic TERRA-interacting proteins screen using SILAC-based quantitative mass spectrometry.^44^ As hSKI was able to bind both ssDNA and ssRNA, we next tested the complex for its binding specificity toward various duplex substrates containing either 3′ or 5′ overhangs that could mimic telomere structures *in vitro* (Figures 4E, 4F, S4A, and S4B). Consistent with its higher affinity for telomeric ssDNA rather than ssRNA, hSKI showed a preference for substrates containing telomeric DNA 3′ overhangs (3′ TEL D:D, K_d_ 6 nM) compared to substrates containing RNA 3′ overhangs (3′ TERRA R:R, K_d_ of 12 nM; 3′ TERRA R:D, K_d_ of 25 nM; Figure 4F). Strikingly, hSKI showed a greater preference for G-rich 3′ overhangs compared to non-telomeric 3′ overhang (Figure 4F, 3′ Non-TERRA-R:D) and poly(A) 3′ overhangs (Figures S4A and S4B). Furthermore, hSKI showed poor binding for structures containing 5′ overhang (5′ TEL D:D and 5′ Non-TERRA R:R) (Figures S4A and S4B). Together, the data indicate that hSKI has preferential affinity for G-rich telomeric substrates harboring 3′ DNA or RNA overhangs, thus advocating a function on telomere structures (G-overhang or DNA-RNA hybrids). Indeed, in a recent study, SKIV2L and TTC37 were found as factors pulled-down using synthetic DNA-RNA hybrids pointing to a possible function mediated by the interaction or regulation of those structures.^45^

### SKIV2L facilitates telomeric DNA-RNA hybrid regulation in G2

**Figure 5:**
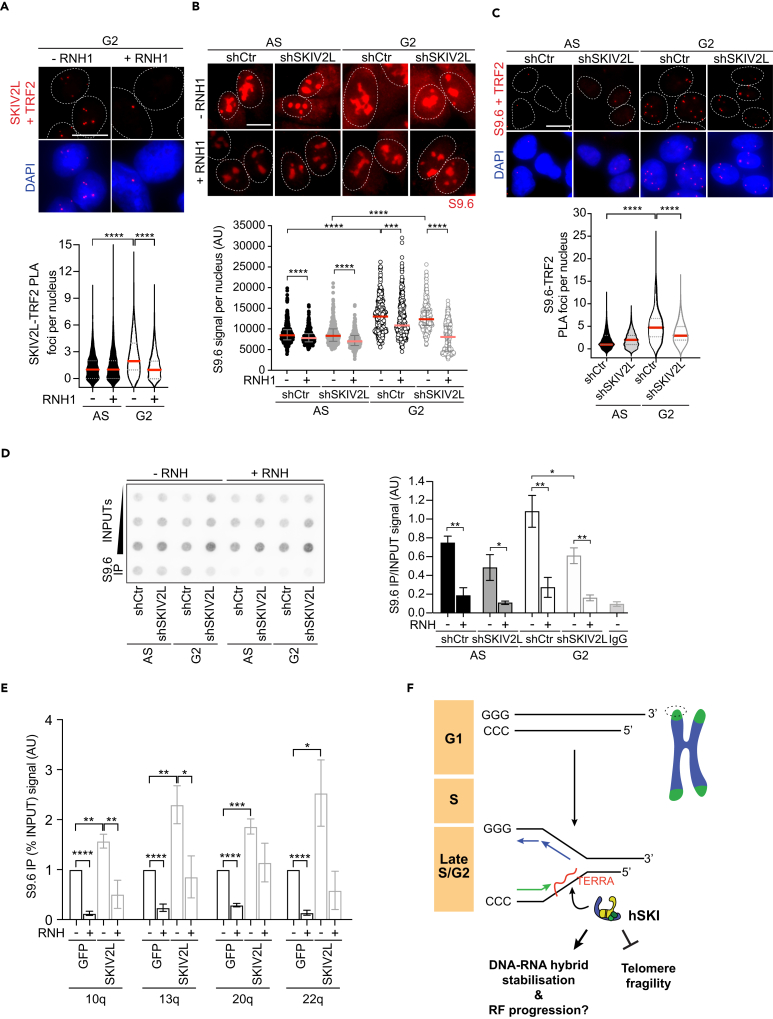
SKIV2L regulates telomeric DNA-RNA hybrids *in cellulo* to prevent telomere fragility (A) Proximity ligation assay (PLA) showing co-localization of SKIV2L and TRF2 in asynchronous (AS) and in G2-synchronized HeLa1.3 cells with and without RNase H1 (RNH1) overexpression (median, Q1 and Q3, at least 600 cells scored per condition, 4 independent experiments, scale bar 10 μm). Mann-Whitney U test ∗∗∗∗*p* < 0.0001. (B) S9.6 IF in HeLa1.3 cells treated with RNAse III, with and without RNase H1 (RNH1) overexpression (median ± interquartile range, 360 cells scored per condition, 4 independent experiments, scale bar, 10 μm). Mann-Whitney U test ∗∗*p* < 0.01, ∗∗∗*p* < 0.001, ∗∗∗∗*p* < 0.0001. (C) PLA showing the co-localization of DNA-RNA hybrids (S9.6) and TRF2 in HeLa1.3 cells pre-extracted and treated with RNAse III (median, Q1 and Q3, at least 400 cells scored per condition, 2 independent experiments, scale bar 10 μm). Mann-Whitney U test ∗∗∗∗*p* < 0.0001. (D) DRIP showing the levels of DNA-RNA hybrids at telomeres in AS and G2-synchronized HeLa1.3 cells, RNH, RNase H treatment (means ± SEM, *n* = 4). (E) DRIP-qPCR assay of G2-synchronized HEK293 cells overexpressing GFP or SKIV2L at 10q, 13q, 20q, and 22q subtelomeric regions. RNH, RNase H treatment (means ± SEM, *n* = 5). Percent input values were normalized to the GFP overexpressing condition. (F) Model proposing the function of hSKI at telomeres. Telomeric DNA-RNA hybrid accumulation in late S/G2 phase drives hSKI recruitment to telomeres to regulate physiological DNA-RNA hybrid levels, prevent telomere replication stress and ensure telomere stability. See also Figure S5.

The biochemistry data indicates that the hSKI complex is able to bind telomeric DNA-RNA hybrids and suggests a possible function *in vivo*, where it could be involved in the metabolism of such molecules at telomeres. Firstly, to corroborate the biochemistry findings and to explore the possible telomeric function of hSKI *in cellulo*, we investigated whether the localization of SKIV2L to telomeres is dependent on the presence of DNA-RNA hybrids. We overexpressed RNase H1-EGFP, which specifically cleaves the RNA moiety of DNA-RNA hybrids^46^ or EGFP alone as a control, in HeLa1.3 cells. We then looked at the co-localization of SKIV2L with TRF2 by PLA (Figures 5A and S5A). We found that RNase H1 overexpression suppresses SKIV2L-TRF2 PLA foci observed in G2-synchronized cells compared to asynchronous EGFP-transfected cells (control). In agreement with the biochemistry data, this result supports that SKIV2L localization to telomeres in G2 is dependent on the presence of telomeric DNA-RNA hybrids.

In order to investigate the possible regulation of DNA-RNA hybrids by SKIV2L, we determined whether its absence perturbs the level of these structures *in cellulo*. We used immunofluorescence of the S9.6 antibody that recognizes DNA-RNA hybrids.^47^^,^^48^ Notably, and in agreement with previous data,^49^ HeLa1.3 cells synchronized in G2 present higher average nuclear S9.6 signal intensity than the asynchronous cell population (Figure 5B, shCtr AS vs*.* shCtr G2), which remained unchanged in SKIV2L-deficient cells synchronized in G2. The average nuclear intensity of DNA-RNA hybrids did not vary drastically in AS SKIV2L-depleted cells compared to AS control cells (Figure 5B, shCtr AS vs*.* shSKIV2L AS). This observation suggests that SKIV2L alone is not sufficient to drastically alter genomic nuclear DNA-RNA hybrids. As expected, overexpression of RNase H1 in all conditions significantly reduced the average S9.6 nuclear signal intensity. Notably, this reduction was much more pronounced in G2 SKIV2L-depleted cells, suggesting that DNA-RNA hybrids are more accessible for RNase H1 processing when SKIV2L is not present.

Having discarded a global effect of SKIV2L on non-telomeric DNA-RNA hybrid levels we tested the effect at telomeres. Telomeric DNA-RNA hybrids can form by hybridization of TERRA molecules with the C-rich telomeric leading strand.^10^^,^^50^ Thus, in order to assess the presence of DNA-RNA hybrids specifically at telomeres, we performed PLA using S9.6 and TRF2 antibodies and measured the resultant co-localization signal (Figure 5C). We observed an increase of PLA foci in G2 cells compared to the asynchronous cell population, in agreement with the increase in global DNA-RNA hybrid signal (Figure 5B) and coincident with a phase of rising TERRA levels after reaching their lowest levels in late S phase/G2 transition in HeLa cells^8^ (Figure S5B). Notably, data showed a significant decrease of PLA foci in SKIV2L-depleted cells synchronized in G2 compared to the control while there was no effect in the asynchronous populations, indicating that G2 telomeric DNA-RNA hybrid levels are reduced in the absence of SKIV2L (Figure 5C). To further support this result, we carried out two independent sets of DNA-RNA immunoprecipitation experiments (DRIP, with sonicated and digested DNA), where the S9.6 antibody is used to precipitate DNA-RNA hybrids, followed by telomere dot-blot. In both experiments, we detected a decrease in the amount of DNA-RNA hybrids at telomeres of cells synchronized in G2 and deficient for SKIV2L compared to AS and control cells (Figures 5D and S5B). Importantly, the signal was abolished in all conditions upon treatment with RNase H, ensuring the specificity of the signal. Altogether, these results support the view that, in cells and particularly at telomeres, SKIV2L contributes to telomere biology by associating with DNA-RNA hybrid structures that are usually formed at long tracks of telomeric repeats.^11^ By binding to telomeres, SKIV2L may help to stabilize or protect telomeric DNA-RNA hybrids.

### SKIV2L helps maintain basal levels of telomeric DNA-RNA hybrids

To further support the hypothesis that SKIV2L protects or stabilize telomeric DNA-RNA hybrids we interrogated the levels of DNA-RNA hybrids upon SKIV2L overexpression. We over-expressed SKIV2L or GFP as a control in G2 synchronized HEK293 cells and carried out DRIP-qPCR^11^^,^^51^ (Figures 5E and S5D). If SKIV2L exerts a protective action over DNA-RNA hybrids we expect that processing factors targeting hybrids would be less efficient upon SKIV2L over-expression, which would lead to DNA-RNA hybrids accumulation. Using subtelomere-specific primers residing in the immediate proximity of 10q, 13q, 20q and 22q-telomere repeats, we analyzed the abundance of DNA-RNA hybrids at these four specific chromosome ends. Telomeric DNA-RNA hybrids signal increased upon over-expression of SKIV2L at the ends of all four chromosomes, which was suppressed by the treatment with RNase H. This indicates that SKIV2L promotes the maintenance of telomeric DNA-RNA hybrids at these telomeres and agrees with the fact that SKIV2L binding to telomeres is dependent on DNA-RNA hybrids and that the DNA-RNA unwinding activity of SKIV2L is dispensable at telomeres.

Considering the binding ability of hSKI to RNAs and TERRA DNA-RNA hybrid structures, we hypothesized that upon SKIV2L deletion, it is the RNA component of DNA-RNA hybrids that might be degraded. We used Northern blotting and RT-qPCR to evaluate the levels of TERRAs. Stable shSKIV2L HeLa1.3 cells showed a slight decrease of long TERRA molecules (Figure S5E, arrow), which was further confirmed by RT-qPCR of TERRAs from chromosomes 10q, 13q, 15q, and 20q (Figure S5F) and to a lesser extend in cells transiently depleted for SKIV2L using siRNA (Figure S5G). These results suggest that SKIV2L helps to maintain telomeric DNA-RNA hybrids by regulating the maintenance of TERRA molecules capable of forming these structures.

Taken together, our results implicate SKIV2L in safeguarding telomere integrity by maintaining a sufficient abundance of physiological basal levels of telomeric DNA-RNA hybrids in G2 of the cell cycle by preventing their degradation or facilitating their processing into functional structures that help maintain telomere stability.

## Discussion

SKI is an essential cofactor of the RNA exosome function in the cytoplasm, which ensures mRNA turnover and quality control^17^^,^^18^^,^^19^^,^^23^^,^^24^ that also localizes in the nucleus and interacts with the PAF complex in humans.^25^^,^^26^ However, and to date, no nuclear function on non-coding RNA processing has been ascribed to the hSKI complex. In this study, we describe a previously unforeseen function for SKIV2L in the nucleus and particularly at telomeres, where the two exclusive components of hSKI, SKIV2L, and TTC37, are enriched during G2, and SKIV2L helps maintain physiological telomeric DNA-RNA hybrid levels, enabling telomere stability before mitosis. In contrast, the impact of the third component of the hSKI complex, WDR61, on telomere stability was not observed, possible due to its pleiotropic function as part of the PAF complex during transcription,^26^ and consistent with its constant binding to telomeres along the cell cycle.

The recruitment of SKIV2L to telomeres coincides with a time (G2) when TERRA levels are rising again after reaching their lowest levels in late S/G2 transition,^8^ and DNA-RNA hybrids are accumulating in HeLa cells. TERRAs can pair with the telomeric C-rich DNA strand generating DNA-RNA hybrids and co-transcriptional R loops. Unscheduled accumulation of these structures in pathological conditions may constitute obstacles for the replication fork and perturb genome stability,^52^^,^^53^ particularly at telomeres^11^^,^^13^ but DNA-RNA hybrids also have a physiological function to regulate telomere biology and stability.^10^^,^^12^^,^^54^^,^^55^ We show that although the global levels of DNA-RNA hybrids are not overly affected in SKIV2L-depleted G2 cells, telomeric DNA-RNA hybrids levels are reduced. This suggests that the nuclear function of SKIV2L is possibly specific to telomeres and extends less to other genomic regions.

Telomeres are difficult-to-replicate regions that resemble fragile sites not only due to their repetitive sequence prone to form secondary structures but the fact that the long telomeric tracks are mainly replicated by replisomes originating in subtelomeric regions.^31^^,^^56^ Our analyses show that SKIV2L suppresses telomere fragility, which supports the hypothesis that SKIV2L has a key role enabling telomeres to deal with replication stress. The role of SKIV2L in facilitating telomere replication is also supported by the fact that SKIV2L loss suppresses aphidicolin-mediated telomere fragility, an effect that was also previously observed in mutants of the CTC1 component of CST complex that promotes efficient replication at telomeres.^33^ This role might be especially relevant at very long tracks of telomeric repeats, where the progression of the replication fork is prone to be hampered and might require the assistance of additional elements to ensure telomere stability before mitosis. In this context, regulation of TERRA biology seems to be essential as supported by the fact that TERRA transcription is required for overcoming telomere replication stress in telomerase positive cells and a number of RNA processing factors being recently isolated at nascent replicating telomeric DNA using QTIP-iPOND.^57^^,^^58^

SKIV2L recruitment to telomeres is dependent on the accumulation of DNA-RNA hybrids in G2, which is also supported by LC-MS/MS-pull-down assays with synthesized DNA-RNA hybrids, in which SKIV2L and TTC37 were identified as DNA-RNA hybrid binding factors.^45^ SKIV2L belongs to the superfamily 2 (SF2) of RNA helicases and has two characteristic RecA-like domains, responsible for nucleic acid binding as well as ATP hydrolysis.^59^ Similar to yeast SKI, hSKI can hydrolyze ATP, an activity that is also essential for its mRNA extraction role.^17^^,^^23^ Our biochemical analysis shows that hSKI binds RNAs including TERRA, however, the affinity is much higher for telomeric DNA, and in particular DNA-RNA hybrids with 3′ telomeric overhangs. However, we propose that the helicase activity of SKIV2L is dispensable for its function at telomeres and SKIV2L acts more as a stabilizer of telomeric DNA-RNA hybrids. It is assumed that RNA helicases unwind DNA-RNA structures *in vitro* due to their ability to displace through the RNA strand. However, many RNA helicases have been involved in DNA-RNA hybrid metabolism by acting through different mechanisms, including UAP56/DDX39B that unwinds harmful R loops genome-wide or DHX9 and UPF1 that, on the contrary, promotes R loop formation at transcription and DSBs sites, respectively, among others.^60^^,^^61^ Interestingly, recent biochemistry analysis of the hSKI complex showed that SKIV2L is able to bind and trap the 80S-ribosome-bound 3′ RNA in a prehydrolytic ATP form closed conformation impeding the release of the gate that protects the 3′ RNA end until hSKI switches to an open conformation.^38^ Upon its binding to telomeres, SKIV2L might bind to 3′ RNA/DNA ends of DNA-RNA hybrid intermediates structures and promote/facilitate their correct processing. This function could be exerted by hSKI alone or in concert with other RNA regulatory factors, including 5′ to 3′ RNA helicase UPF1, hEST1A or SMG1, all involved in telomere length homeostasis by negatively regulating TERRA association with telomeres.^5^^,^^16^ Our model is supported by recent evidence demonstrating that the protection/stabilization of DNA-RNA hybrids at telomeres is essential to guarantee specific telomere functions. Recently, RTEL1 DNA helicase was similarly reported to be involved in either facilitating the formation or maintaining TERRA-containing telomeric DNA-RNA hybrids and promoting telomere stability.^14^ A similar role promoting telomeric hybrid stabilization is exerted by some RNA binding proteins in yeast such as Npl3, which binds exclusively short telomeres to promote their elongation by homology directed repair.^15^ Additionally, helicase UPF1 was reported to facilitate the formation of DNA-RNA hybrids at DSBs induced at subtelomeric regions to stimulate DSB repair.^62^

In agreement with previous reports showing increased levels of DNA-RNA hybrids in G2 of the cell cycle,^49^^,^^63^ our results also suggest that telomeres accumulate more DNA-RNA hybrids in G2, that mediates the recruitment of additional factors, including SKIV2L. The physiological function of those hybrids in G2 remains to be investigated. They could be a prerequisite for mitotic telomere condensation, considering the strong correlation between DNA-RNA hybrids and chromatin compaction marks in both yeast and human cells, and their importance for the establishment of repressive heterochromatin at mammalian gene terminators.^53^^,^^64^^,^^65^^,^^66^ Alternatively, they could be transient intermediates resulting for the processing of stalled replication forks at long difficult-to-replicate telomeric repeats, which is supported by the fact that several DNA replication factors have been isolated as TERRA interacting factors.^54^ The equilibrium among dsDNA and DNA-RNA hybrid structures could favor the affinity of different regulatory factors to telomeres that ensure telomere stability, including SKIV2L. Interestingly, this kind of regulation has been recently uncovered for the DNA methyltransferase 1 (DNMT1) at gene promoters.^67^ DNMT1 binding affinity to DNA-RNA hybrid structures is reduced compared to dsDNAs, thus decreasing promoter methylation and promoting transcription at R loop-enriched regions. We cannot exclude that a similar regulation, mediated by SKIV2L or other RNA processing factors, might occur at human telomeres so that it would affect the activity of the subset of TERRA promoters containing CpG islands, which are regulated by both DNMT1 and DNMT3b enzymes.^68^^,^^69^

We propose a model in which hSKI safeguards a fraction of telomeres by being recruited to telomeres in G2 phase of the cell cycle in a DNA-RNA hybrid-dependent manner (Figure 5F). According to our model, the transient formation of DNA-RNA hybrids at long tracks of telomeric repeats favors hSKI binding, which enables the stabilization of the transient telomeric DNA-RNA hybrids forming in G2. Once there, SKIV2L facilitates the correct processing of DNA-RNA hybrid structures and prevent the accumulation of aberrant structures, which may hinder DNA replication and result in increased telomere fragility.

In summary, this study demonstrates that human SKIV2L of the SKI complex, known for its cytoplasmic role in NMD, is also a chromatin telomeric factor. In particular, SKIV2L associates with telomeres in G2 and ensures telomere stability before mitosis. The regulation of telomeric lncRNAs (TERRAs) and DNA-RNA hybrids is essential for telomere homeostasis and cells use different components of the RNA processing machineries, including SKIV2L to achieve chromatin compaction, cell cycle regulation, and genome stability. Further efforts will be necessary to fully understand the complexity and regulation of the different factors involved in telomeric RNA processing along with the human SKI complex at telomeres.

### Limitations of the study

While SKIV2L and TTC37 clearly show the formation of foci in the nucleus, most of the nuclear SKIV2L is non telomeric, and most telomeres do not bind SKIV2L at the same time. The G2 specific increase reported at telomeres could be a consequence of increased SKIV2L binding to chromatin in G2.

## Resource availability

### Lead contact

Further information and requests for resources and reagents should be directed to and will be fulfilled by the Lead Contact, Jean-Baptiste Vannier (j.vannier@lms.mrc.ac.uk).

### Materials availability

All unique/stable reagents generated in this study are available on request from the lead contact.

### Data and code availability

• The data underlying this article have been deposited at Zenodo (https://zenodo.org/uploads/10015203) and will be shared by the lead contact upon request. FACS data have been deposited at FLOWRepository and are publicly available as of the date of publication. PICh mass spectrometry data have been deposited at ProteomeXchange and are publicly available as of the date of publication.
• DOIs and accession numbers are listed in the key resources table.
• This study does not report original code.
• Any additional information required to reanalyze the data reported in this paper is available from the lead contact upon request.

## Acknowledgments

Vannier lab’s work is supported by the London Institute of Medical Sciences (LMS), which receives its core funding from 10.13039/100014013UKRI (MRC), by an ERC Starter grant (637798; MetDNASecStr) and is thankful to Imperial College London for its support. Roser Gonzalez-Franco received an MRC funded PhD fellowship, Jean-Baptiste Vannier and Lepakshi Ranjha are funded by 10.13039/100014013UKRI and Imperial College London. Emilia Herrera-Moyano, Rosa Maria Porreca, Eleni Skourti are funded by ERC Starter grant (637798; MetDNASecStr). Manos Stylianakis is funded by an MRC PhD fellowship. The revision work for this manuscript was carried away by Emilia Herrera-Moyano and funded by a grant from the 10.13039/501100011033Spanish Agencia Estatal de Investigación (AEI; PID2022-138251NB-I00 funded by 10.13039/501100004837Ministerio de Ciencia e Innovación/AEI/10.13039/501100011033; “ERDF: a way of making Europe”). Special thank you to Andrés Aguilera and Aguilera's laboratory (CABIMER, Universidad de Sevilla) for highly constructive discussions and for hosting the revision. We thank Titia de Lange for providing HeLa1.3 cells, Petr Cejka for Sf9 insect cells, Joachim Lingner for HT1080-ST cells and Robert J Crouch for RNase H1-overexpressing plasmids.

## Author contributions

E.H.M., R.M.P., L.R., R.G.F., E.S., and J.B.V. designed the project and wrote the manuscript. Molecular biology experiments were carried out by E.H.M., R.M.P., R.G.F., E.S., R.L., and M.S. Biochemistry was conducted by L.R. and Y.S. A.M. and H.K. performed mass spectrometry running and primary analysis. A.A.S. worked on the structural analysis. E.H.M. and J.B.V. were responsible of the visualization and curation of the data and conceived, reviewed and edited the final manuscript. J.B.V. supervised the work.

## Declaration of interests

The authors declare no conflict of interests.

## STAR★Methods

### Key resources table

**Table:** 

REAGENT or RESOURCE	SOURCE	IDENTIFIER
**Antibodies**		

Rabbit polyclonal anti-SKIV2L	Proteintech group	Cat#11462-1-AP; RRID:AB_2187472
Rabbit polyclonal anti-TTC37	Novus Biologicals	Cat#NBP1-93640; RRID:AB_11030277
Rabbit polyclonal anti-WDR61	Sigma-Aldrich	Cat#SAB1401852; RRID:AB_10609719
Rabbit polyclonal anti-WDR61	Thermo Fisher Scientific	Cat#PA5-40079; RRID:AB_2605542
Mouse monoclonal anti-human RNASEH1 (Clone 5D10)	Abnova	Cat#H00246243-M01; RRID:AB_530236
Rabbit polyclonal anti-GFP	Abcam	Cat#ab290
Mouse monoclonal anti-beta-Actin	Abcam	Cat#ab8226; RRID:AB_306371
Mouse monoclonal anti-Histone H3	Abcam	Cat#ab10799; RRID:AB_470239
Mouse monoclonal anti-alpha-Tubulin	Sigma-Aldrich	Cat#T6199
Rabbit polyclonal anti-TRF2	Novus Biologicals	Cat#NB110-57130; RRID:AB_844199
Mouse monoclonal anti-TRF2 (Clone 4A794)	Millipore	Cat#05-521; RRID:AB_2303145
Mouse monoclonal anti-TRF1	Santa Cruz Biotechnology	Cat#sc-56807; RRID:AB_793407
Mouse monoclonal anti-TIN2	Sigma-Aldrich	Cat#SAB4200108; RRID:AB_10624591)
Mouse monoclonal anti-DNA-RNA Hybrid	Kerafast	Cat#ENH001, RRID:AB_2687463
Mouse monoclonal anti-Phospho-ATM (Ser1981) (10H11.E12)	Cell Signaling	Cat#4526
Rabbit polyclonal anti-53BP1	Novus Biologicals	Cat#NB 100-304, RRID:AB_350221
Mouse monoclonal anti-Cyclin A (At10.2)	Santa Cruz biotechnology	Cat#sc-53227; RRID:AB_782329
Goat anti-rabbit Alexa Fluor 594	Thermo Fisher Scientific	Cat#A-11037
Goat anti-mouse Alexa Fluor 594	Thermo Fisher Scientific	Cat#A-11005
Donkey anti-rabbit Alexa Fluor 488	Thermo Fisher Scientific	Cat#A-21206
Donkey anti-mouse Alexa Fluor 488	Thermo Fisher Scientific	Cat#R37114
goat anti-rabbit Alexa Fluor 488	Thermo Fisher Scientific	Cat#A11008
Goat anti-Mouse Ig/HRP	Agilent	Cat#P0447, RRID:AB_2617137
Swine anti-rabbit Ig/HRP	Agilent	Cat#P0217, RRID:AB_2728719
anti-DIG-alkaline phosphatase	Roche	Cat#11093274910

**Bacterial and virus strains**		

*E. coli*: Stable Competent cells	New England BioLabs	Cat#C3040H

**Chemicals, peptides, and recombinant proteins**		

Aphidicolin	Sigma-Aldrich	Cat#A0781
Thymidine	Sigma-Aldrich	Cat#T1895
Nocodazole	Sigma-Aldrich	Cat#M1404
Phosphatase inhibitor	Sigma-Aldrich	Cat#P0044
RNase III	Thermo Fisher Scientific	Cat#AM2290
Colcemid	Roche	Cat#10295892001
RNase H	New England BioLabs	Cat#M029L
RNase A	Roche	Cat#10109169001
EdU	Thermo Fisher Scientific	Cat#E10415
7-Amino-Actinomycin D	BD Biosciences	Cat#559925
Propidium Iodide	Sigma-Aldrich	Cat#P4864
ProLong gold antifade Mountant with DAPI	Invitrogen	Cat#P36935
Benzonase	Sigma-Aldrich	Cat#E1014-25KU
cOmplete, EDTA-free Protease Inhibitor Cocktail	Roche	Cat#11873580001
Protease inhibitor cocktail	Sigma-Aldrich	Cat# P8340
DNase I	Qiagen	Cat#79254
DNase I	Roche	Cat#4716728001
SuperScript III Reverse Transcriptase	Thermo Fisher Scientific	Cat#18080093
Fetal Bovine Serum	Sigma-Aldrich	Cat#F2442
Insect-XPRESS medium	Lonza	Cat#BELN12-730Q
Lipofectamine RNAiMax	ThermoScientific	Cat#13778150
Normal Donkey serum	Jackson ImmunoResearch	Cat#017-000-121
Normal Goat serum	Jackson ImmunoResearch	Cat#005-000-121
Blocking Reagent	Roche	Cat#11096176001
Dynabeads Protein A	Invitrogen	Cat#10001D
Dynabeads Protein G	Invitrogen	Cat#10004D
CDP-Star ready-to-use	Roche	Cat#12041677001
BsrGI-HF	New England Biolabs	Cat#R3575S
EcoRI-HF	New England Biolabs	Cat#R3101S
HindIII-HF	New England Biolabs	Cat#R3104S
SspI-HF	New England Biolabs	Cat#R3132L
XhoI	New England Biolabs	Cat#R0146L
Glycogen	Thermo Fisher Scientific	Cat#R0561
Ni-NTA agarose	Qiagen	Cat#30210
Bio-Rad Protein Assay Dye Reagent Concentrate	Bio-Rad	Cat#500-0006
Trypsin Gold, Mass Spectrometry Grade	Promega	Cat#V5280
UltraHyb-Oligo solution	Thermo Fisher Scientific	Cat#AM8663

**Critical commercial assays**		

3’ end labelling kit	Roche	Cat#03353575910
RNeasy Mini Kit	Qiagen	Cat#74104
iTaq Universal SYBR Green Supermix	Bio-Rad	Cat#1725124
Power SYBR Green PCR Master Mix	Applied Biosystems	Cat#4368708
Click-iT EdU Alexa Fluor 647 Flow Cytometry Assay kit	Thermo Fisher Scientific	Cat#C10634
Click-iT Edu Alexa Fluor 647 Imaging Kit	hermo Fisher Scientific	Cat#C10640
Click-iT EdU Alexa Fluor 488 Imaging kit	Thermo Fisher Scientific	Cat#C10337
Duolink PLA Red Starter Kit	Sigma-Aldrich	Cat#DUO92101
Nucleofector kit R	Lonza	Cat#VVCA-1001
Nucleofector Kit V	Lonza	Cat#VVCA-1003
QuickChange Lightning Multi Site-Directed Mutagenesis Kit	Aligent Technologies	Cat##210513

**Deposited data**		

FACS data for cell cycle synchronisation and analysis HeLa1.3 cells	This paper, FLOWRepository	FR-FCM-Z6UL (http://flowrepository.org/id/RvFrbTWARY1y21uJW9H6F6kRo6g2gxddtnRXZHYLwP0vvYBl5OSEapEbgElyn0rp)
FACS data for cell cycle analysis of HeLa1.3 shCtr or SKIV2L depleted treated with DMSO or APH	This paper, FLOWRepository	FR-FCM-Z6UR (http://flowrepository.org/id/RvFrjD1JJzAVyuLfZU1I3VYSQjN1gkLsX1swjnyfnv80eupejavnlAbSdgWHB7Ws)
FACS data for cell cycle analysis HEK293 cells	This paper, FLOWRepository	FR-FCM-Z6UX (http://flowrepository.org/id/RvFr42D68NSnAwxaQDdBbxD7uq9CmVKK6QxqtXNKvuT0Qw0sJk18HtoHxnbaVhIM)
Raw and underlying data	This paper, Zenodo	https://doi.org/10.5281/zenodo.10015203
PICh mass spectrometry data	This paper, ProteomeXchange	https://www.proteomexchange.org/Accession number: PXD046955

**Experimental models: Cell lines**		

*Spodoptera frugiperda*: Sf9 insect cells	Laboratory of P. Cejka	N/A
Human: HeLa1.3 cells	Laboratory of T. De Lange. https://doi.org/10.1074/jbc.M109.038026	N/A
Human: HT1080-ST cells	Laboratory of J. Lingner. https://doi.org/10.1038/sj.emboj.7600952	N/A
Human: HEK293FT cells	ATCC	N/A
Human: U2OS cells	ATCC	Cat#HTB-96
Human: IMR90 cells	Coriell Institute	Cat#I90-83

**Oligonucleotides**		

ON-TARGETplus Non-targeting Pool siRNA	Dharmacon	Cat#D-001810-10-20
SMARTpool: ON-TARGETplus SKIV2L siRNA	Dharmacon	Cat#L-013435-01-0010
SMARTpool: ON-TARGETplus TTC37 siRNA	Dharmacon	Cat#L-020959-02-0020
Set of 4: ON-TARGETplus WDR61 siRNA	Dharmacon	Cat#LQ-014614-01-0002
Primers for qPCR, see Table S3	This paper, Feretzaki et al.^70^, Sagie et al.^11^, Porro et al.^8^	N/A
Oligonucleotides used for EMSA assays, see Table S2	This paper	N/A
Tel-Cy3-O-O-(CCCTAA)3 probe for FISH	PNA bio	Cat#F1002
Telomeric C-rich probe: TAA(CCCTAA)_4_	This paper	N/A
Telomeric G-rich probe: ATT(GGGATT)_4_	This paper	N/A

**Recombinant DNA**		

pLKO.1 SKIV2L shRNA	Dharmacon	RNAi Consortium TRCN0000051816
pEGFP	Laboratory of RJ. Crouch, Cerritelli et al.^71^	N/A
pEGFP-M27-H1	Laboratory of RJ. Crouch, Cerritelli et al.^71^	N/A
pcDNA3.1(+)-GFP	GenScript	N/A
pcDNA3.1(+)-SKIV2L	GenScript	N/A
pcDNA3.1(+)-SKIV2L (siRes)	This paper	N/A
pcDNA3.1(+)-SKIV2L-V341G	This paper	N/A
pcDNA3.1(+)-SKIV2L-K338R	This paper	N/A
pcDNA3.1(+)-SKIV2L-D243R	This paper	N/A
pFastbac1	GenScript	N/A
pFB-SKIV2L	This paper	N/A
pFB-His-Flag-TTC37	This paper	N/A
pFB-WDR61	This paper	N/A
pFB-MBP-SKIV2L	This paper	N/A

**Software and algorithms**		

Fiji	https://doi.org/10.1038/nmeth.2019	https://imagej.net/software/fiji/
ImageJ macro code used for quantification of S9.6 signal intensity per nucleus	This paper, Zenodo	https://zenodo.org/doi:10.5281/zenodo.10015203
Image Studio Lite 5.0	LICORbio	https://www.licor.com/bio/image-studio/
ImageQuant	Cytiva	https://info.cytivalifesciences.com/image-analysis-software.html
PyMOL	Schrödinger	https://www.pymol.org/
ChimeraX	UCSF Resource for Biocomputing, Visualization, and Informatics, Pettersen et al.^72^	https://www.cgl.ucsf.edu/chimerax/
MaxQuant software platform (v1.5.8.3)	Max Planck Institute of Biochemistry	https://www.maxquant.org/
GraphPad Prism 9.1.0	Dotmatics	https://www.graphpad.com/
Carl ZEISS ZEN 2 software (blue edition) version 2.0.0.0	ZEISS	https://www.micro-shop.zeiss.com/

### Experimental model and study participant details

#### Cell lines

Stable clone of adult female human cervical adenocarcinoma HeLa cell line with long telomeres (HeLa1.3 cells); adult male human fibrosarcoma HT1080 cell line overexpressing telomase (HT1080 super-telomerase cells, HT1080-ST) (kindly provided by T. de Lange and J. Lingner, respectively); adult female human osteosarcoma U2OS cell line (ATCC); human embryonic kidney HEK293FT cell line (ATCC); and human female fetal lung fibroblast IMR90 cell line (Coriel Institute) were cultured in DMEM medium supplemented with 10% (v/v) fetal bovine serum (FBS, Sigma-Aldrich, F2442) and maintained at 37°C in 5% (v/v) CO_2_. *Spodoptera frugiperda* Sf9 insect cells (kindly provided by P. Cejka) were grown at 27°C in serum free Insect-XPRESS medium (Lonza, BELN12-730Q). Cells lines derived from ATCC are authenticated before the purchase by short tandem repeat analyses. Comprehensive quality-control tests based on the optical observation of cellular morphology, evaluation of cell proliferation and mycoplasma detection were routinely performed.

### Method details

#### Generation of stable cell lines

For the generation of SKIV2L and CONTROL knockdown HeLa1.3 and HEK293 stable cells, lentiviral particles were produced in HEK293FT cells using pLKO.1 SKIV2L shRNA construct (Dharmacon, 5'-GTACACTATGATCCTCAACTT-3', RNAi Consortium TRCN0000051816) and pGIPZ (Control, Ctr) constructs. Cells were transduced following standard procedures. Selection and clonal population generation was achieved with 1 μg/ml puromycin. Cells expressing the shRNA were maintained in 0.25 μg/ml puromycin-containing media.

#### DNA plasmids and transfections

For the overexpression of RNase H1 we used pEGFP-M27-H1 and pEGFP as a control (kindly gifted to us from RJ. Crouch).^71^ Their transient transfection into HeLa1.3 cells was performed using the Nucleofector kit R (Lonza #VVCA-1001) according to the manufacturer’s instructions. Briefly, 4 million HeLa1.3 cells were transfected with 12 μg of plasmid DNA (in a final volume of 100 μl in Nucleofector solution (82 μl) mixed with supplementary solution (18 μl) supplied in the kit. Nucleofector™ II/2b Device (Lonza #LO AAB-1001) programme A-020 was used. Cells were seeded into 1 x 10 cm dishes for each condition and collected after 24 h for downstream analysis. For the overexpression of SKIV2L, pcDNA3.1(+)-SKIV2L and pcDNA3.1(+)-GFP (control) plasmids were generated by GenScript. For the production of a shRNA resistant version of SKIV2L plasmid (pcDNA3.1(+)-SKIV2L (siRes)), changes were introduced in SKIV2L using primer 5'-CAGCTGCAGTCCCAGTTCCGCCTCACGTATACGATGATTTTGAATCTGCTGCGAGTGGATGC-3' and QuickChange Lightning Multi Site-Directed Mutagenesis Kit (Aligent Technologies) according to the manufacturer’s instructions. pcDNA3.1(+)-SKIV2L-V341G, carrying 1022T>G missense mutation was produced by site-directed mutagenesis of pcDNA3.1(+)-SKIV2L (siRes) using same kit and the 5’-CTGCAGGAAAAACAGTTGGGGCTGAATATGCCATTGC-3’ primer. Similarly, K338R and D243R mutants were obtained with respectively: 5'-CTCACACATCTGCAGGAAGAACAGTTGTGGCTGAAT-3' and 5'-CTGGAGTGGGTCATCTTTGCTGAGGTTCACTATATCAAC-3' primers. Plasmids were transfected into HEK293FT cells using the Nucleofector Kit V (Lonza #VVCA-1003) following the method described for HeLa1.3 cells and the program Q-001.

#### siRNA-mediated depletion

For the siRNA transfections, 1 million HeLa1.3 or HEK293 cells, 400 thousand HT-1080-ST cells or 500 thousand IMR90 cells were seeded per 10 cm dish 5 h before transfection. Cells were transfected with 15 μl of 20 μM siRNA (SMARTpool: ON-TARGETplus SKIV2L siRNA (Dharmacon #L-013435-01-0010), SMARTpool: ON-TARGETplus TTC37 siRNA (Dharmacon #L-020959-02-0020), Set of 4: ON-TARGETplus WDR61 siRNA (Dharmacon #LQ-014614-01-0002) or ON-TARGETplus Non-targeting Pool (Dharmacon #D-001810-10-20)) using 30 μl of Lipofectamine RNAiMax transfection agent (ThermoScientific #13778150) according to manufacturer’s instructions in a final volume of 3 ml (final siRNA concentration: 100 nM). Transfection was repeated 72 h later. 72 h after the second transfection, cells were harvested for downstream analysis at this time point.

#### Cell synchronisation and drug treatments

HeLa1.3 cells were treated with 2.5 mM thymidine (Sigma-Aldrich, T1895) for 24 h, then washed and allowed to grow in normal media for 16 h followed by a second incubation with 2.5 mM thymidine for 24 h. Cells were released from the block and collected at the specified time points. S-phase or G2-phase enriched cell populations were released for 4 h and 7 h, respectively. For enrichment of mitotic cells, HeLa1.3 were initially treated with 2.5 mM thymidine for 16 h. After a release for 8 h in fresh media they were incubated with 50 ng/mL nocodazole (Sigma-Aldrich, M1404) for 16 h. Cells were collected with no release period. When indicated, HeLa1.3 cells were treated with 0.2 μM aphidicolin for 20 h or with the same amount of DMSO (untreated control). If indicated, transfection of synchronized G2 HeLa1.3 cells (in parallel with asynchronous HeLa1.3) was performed as described above in the ‘DNA plasmids and Transfection’ section after the first thymidine block, coinciding with the beginning of the first release. HEK293FT cells were treated with 2.5 mM thymidine for 24 h, then washed, released from the block and collected at the specified time points. G2-phase enriched cell population was released for 6 h. Transfections were performed 6 hours before the thymidine block.

#### Fractionation assay

HeLa1.3 cells were scraped in ice-cold CSK buffer (10 mM PIPES KOH pH 6.8, 100 mM NaCl, 300 mM Sucrose, 1.5 mM MgCl2, 5 mM EDTA, 0.5% (v/v) Triton-X-100, cOmplete, EDTA-free Protease Inhibitor Cocktail 2X (Roche, 11873580001), phosphatase inhibitor (Sigma-Aldrich, P0044), homogenised and incubated on ice for 10 min. The 1/3 was kept as the whole cell extract. The rest of the sample was centrifuged for 3 min at 3000 rpm. The supernatant was collected as the soluble fraction, whilst the pellet was washed once with CSK buffer and resuspended in lysis buffer. This was homogenised with a 0.5 mm needle and incubated at room temperature for 20 min before snap freezing. α-Tubulin (cytoplasmic) and H3 (chromatin-binding) were used as controls.

#### Immunofluorescence (IF)

Cells were grown on 4-well culture slides or on coverslips, pre-extracted for 5 min with permeabilisation buffer (50 mM NaCl, 3 mM MgCl2, 20 mM Tris pH 8, 0.5% (v/v) Triton X-100, 300 mM sucrose), fixed for 15 min in fixative solution (formaldehyde 4% (w/v) / sucrose 2% (w/v)) and permeabilised for another 10 min. Slides were incubated for 30 min with the blocking buffer (10% (v/v) goat or donkey serum (Jackson ImmunoResearch) in 1X PBS) at 37°C. For 53BP1 IF, slides were incubated for 1 h with blocking buffer (3% BSA). Then, the primary antibody (anti-SKIV2L, Proteintech group, 11462-1-AP, 1:1000; anti-TTC37, Novus Biologicals, NBP1-93640, 1:500; anti-TRF2, Millipore, 05-521, 1:500; anti-TRF2, Novus Biologicals, NB110-57130, 1:500, anti-ATM pS1981, Cell signalling, 4526, 1:500 or anti-53BP1, Novus Biological, NB100-304, 1:500) was added in blocking buffer and incubated for 1 hour at 37°C. After 3 x 3 min washes with 1X PBS, the secondary antibody (1:400 in blocking buffer, goat anti-rabbit Alexa 594, Thermo Fisher Scientific, A-11037; donkey anti-rabbit Alexa 488, Thermo Fisher Scientific, A-21206, donkey anti-mouse Alexa 488, Thermo Fisher Scientific, R37114 or goat anti-rabbit Alexa 488, Thermo Fisher Scientific, A11008, 1:1000 for 53BP1) was added in blocking buffer for 30 min at 37°C, followed by 3 x 3 min washes with PBS 1X. Finally, mounting media containing DAPI (ProLong gold antifade Mountant with DAPI, Invitrogen, P36935) was used to counterstain DNA.

#### S9.6 IF

S9.6 IF was performed as previously described^48^^,^^49^ with minor modifications. Briefly, cells were pre-extracted for 3 min using ice-cold pre-extraction buffer (0.5% Triton X-100, 20 mM HEPES-KOH (pH7.9), 50 mM NaCl, 3 mM MgCl2, and 300 mM sucrose) and fixed and permeabilised as described above. Before the immunostaining, a treatment with Ambion RNase III (1.2 U, Thermo Fisher Scientific, AM2290) for 30 min at 37°C was performed, to remove dsRNAs that could interfere with the staining. Cells were blocked for 1 h in blocking buffer (BSA 3% (w/v) in PBS), incubated with the mouse anti-DNA-RNA hybrid S9.6 antibody (1/500, Kerafast ENH001) in blocking buffer overnight at 4°C, followed by 3 washes with PBS and detection using Alexa Fluor 594 goat anti-mouse secondary antibody (1:800 in blocking buffer) for 1 h at 37°C. Slides were imaged using a Zeiss microscope using Carl Zeiss software. The averages of S9.6 signal intensity per nucleus (A.U., Arbitrary Units) were quantified using the ImageJ software (Fiji).

#### Proximity Ligation Assay (PLA)

For proximity ligation assay experiments, HeLa1.3 cells were cultured on glass coverslips and pre-extracted, fixed and permeabilised as described above. For S9.6 PLA, samples were treated with RNase III as described above. PLA was performed following the manufacturer’s instructions (Duolink DUO92101, Sigma-Aldrich) with some modifications. For S9.6-TRF2 PLA, cells were blocked for 3 h (1 h at 37°C and 2 h at 4°C) in blocking buffer (BSA 2% (w/v) in PBS), incubated with the mouse anti-DNA-RNA hybrid S9.6 antibody (1/2000, Kerafast ENH001) and rabbit anti-TRF2 antibody (1/1000, Novus NB110-57130) in blocking buffer overnight at 4°C followed by 3 washes with PBS-Tween 0.1% (v/v). For TRF2-SKIV2L PLA, IF was performed as described above using anti-SKIV2L (Proteintech group, 11462-1-AP, 1:500) and anti-TRF2 (Millipore, 05-521, 1:500) primary antibodies. For detection, anti-Mouse minus and anti-Rabbit plus PLA probes were incubated in blocking buffer for 1 h at 37°C and ligation and amplifications reactions were performed for 30 and 100 min respectively at 37°C. Slides were imaged as described above. Control reactions using only one of the antibodies in each case were performed. The number of foci per nucleus were manually counted using the ImageJ software (Fiji).

#### Quantitative-FISH analysis (Q-FISH)

For metaphase preparation, HeLa1.3, HT1080-ST or U2OS cells were incubated for 1 h and IMR90 cells for 4 hours with colcemid 10 ng/ml (Roche, 10295892001). Subsequently, cells were collected and incubated at 37°C in hypotonic buffer (HeLa1.3 and IMR90: 15 and 10 min, respectively, KCl 75 mM: NaCitrate 8g/L: ddH2O, 1:1:1 and HT1080-ST and U2OS: 40 min and 12 min respectively, NaCitrate 8 g/L). Fixation was performed with freshly prepared fixative (ethanol: glacial acetic acid (3:1)) followed by three washes using the same fixative. Finally, metaphase suspensions were dropped on previously humidified glass slides. Q-FISH was performed as previously described.^73^ Briefly, metaphase spreads were fixed in formaldehyde 4% (w/v) for 2 min, washed 3 x 5 min in PBS 1X, treated with pepsin (1 mg/ml in 0.05 M citric acid pH 2) for 10 min at 37°C, post-fixed for 2 min, washed and incubated in increasing ethanol concentration baths. Each slide was then covered with hybridising solution containing Cy3-O-O-(CCCTAA)3 probe (PNA bio) in formamide 70% (v/v), 10 mM Tris pH 7.4 and 1% (v/v) blocking reagent (Roche, 11096176001). This step was followed by denaturation for 3 min at 80°C on a heat block. Hybridisation was performed for 2 h at room temperature. Slides were washed 2 x 15 min in formamide 70% (v/v) in 20 mM Tris pH 7.4, followed by 3 x 5 min washes in 50 mM Tris pH 7.4, 150 mM NaCl, Tween-20 0.05% (v/v), dehydrated in successive ethanol baths and air-dried. Slides were mounted in antifade reagent containing DAPI and imaged as described above. Telomeric signals were quantified using the ImageJ software (Fiji). Striped, abnormal elongated signal or multi-telomeric signals detected in one chromosome end was considered as a fragile telomere and the absence of telomeric signal as telomere loss. The percentage of fragile telomeres or telomere loss versus the total number of chromosome ends was calculated.

#### IF-FISH

For IF-FISH experiments, slides were post-fixed using fixative solution (formaldehyde 4% (w/v) / sucrose 2% (w/v)) after secondary antibody and PBS washes treatments. FISH was then performed as in metaphases, using a Cy3-O-O-(CCCTAA)_3_ probe (PNA bio). For 53BP1-Telomere FISH experiment, slides were imaged using a Leica TCS SP5 confocal microscope.

#### Chromatin immunoprecipitation (ChIP)

Chromatin preparation and ChIP experiments were performed as previously described^74^ with the following modifications: Chromatin (50 μg) was diluted in 1 ml final volume of ChIP dilution buffer (20 mM Tris-HCl pH 8, 150 mM KCl, 2 mM EDTA pH 8, 1% (v/v) Triton X-100, 0.1% (w/v) SDS), pre-cleared for 2 h with Dynabeads Protein A (Invitrogen, 10001D), followed by overnight incubation with 5 μg of antibody (anti-SKIV2L Proteintech group, 11462-1-AP; anti-TRF1 Santa Cruz Biotechnology, sc-6165-R). DNA was incubated in 0.4 M NaOH 10mM EDTA and denatured at 100°C for 10 min. Finally, DNA was spotted onto a positively charged Amersham Hybond N+ membrane (GE Healthcare, RPN303B) using a dot-blot apparatus. Membranes were UV-crosslinked (Stratalinker, 200 J/cm^2^ UV), heated for 1 h at 100°C and hybridised overnight at 42°C with the telomeric C-rich digoxigenin (DIG)-labeled probe TAA(CCCTAA)_4_. The probes were prepared using 3’ end labelling kit (Roche, 03353575910) according to the manufacturer’s instructions and diluted in hybridisation buffer (6X SSC, 0.1% SDS, 1% milk). The membrane was then washed 2 x 5 min with pre-warmed 2X SSC/0.1% SDS and 1 x 2 min with pre-warmed 0.2X SSC/ 0.1% SDS, then submerged in 2X SSC for 5 min and washed in Tween-20 in maleic acid 0.3% (v/v) for 5 min. Signal was detected using the anti-DIG-alkaline phosphatase antibodies (1:20000, Roche, 11093274910) and CDP-Star ready-to-use (Roche, 12041677001) following the manufacturer’s instructions. Images were captured using the Amersham Imager 680 (GE Healthcare) and analysed using the Image Studio Lite software.

#### DNA-RNA immunoprecipitation (DRIP)

DRIP experiments were performed as previously described with the following modifications.^11^^,^^75^^,^^76^ Total nucleic acids, gently extracted using phenol-chloroform from one 15-cm dish of HeLa1.3 cells growth to 75-80% confluency, were digested using BsrGI, EcoRI, HindIII, SspI and XhoI restriction enzymes (New England Biolabs, 44 U each). Digested nucleic acids were EtOH-precipitated with 1.5 μl of glycogen (Thermo Fisher Scientific, R0561) and gentle resuspended in 50 μl of 1x TE buffer. 8 μg of digested nucleic acids were treated or not with 10 μl of RNase H (New England Biolabs, M029L) overnight at 37°C in 1x RNAse H buffer and 1/10 of the samples were used as input. Samples were incubated with 20 μl of the S9.6 antibody (Kerafast ENH001) for 14-17 h at 4°C followed by the incubation with 100 μl of Dynabeads Protein A + G (2:1 A:G proportion, Invitrogen, 10001D and 10004D) for 2 h at 4°C. Then, precipitated samples were eluted in 300 μl of elution buffer (50 mM Tris, pH 8.0, 10 mM EDTA, pH 8.0, 0.5% (v/v) SDS), treated with 5.8 μl of proteinase K (24 mg/mL, P4850) for 45 min at 55°C followed by 50 μg/ml RNase A (Roche, 10109169001) for 1 h at 37°C plus 1 h at 65°C. Finally, cleaned samples were resuspended in 100 μl 1X TE buffer and blotted using a dot-blot apparatus. Probe preparation and membrane detection were performed as described in the ChIP section. Telomeric G-rich probe ATT(GGGATT)_4_ was used for the detection of the precipitated DNA. In HEK293 cell experiments input and precipitated DNA were detected using qPCRs at the indicated subtelomeric regions with the corresponding primers listed in Table S3.^11^^,^^68^ qPCR was performed with 2 μl of immunoprecipitated DNA and 2 μl of a 1/25 dilution of input samples in a final volume of 20 μl using the iTaq Universal SYBR Green Supermix (Bio-Rad, 1725124) and analysed on 7500 FAST Real-Time PCR system (Applied Biosystems). 40 cycles of 3 s of denaturation at 95°C followed by 30 s of annealing and extension at 60°C were used. The percent input method was used for relative quantification and normalised to the pEGFP control signal.

#### Additional DRIP experiments

Additional DRIP experiments were performed using sonicated nucleic acids.^13^ Samples were sonicated using Bioruptor (Diagenode) to obtain around 500 bp long fragments and 30 μg of digested nucleic acids were treated or not with 10 μl of RNase H overnight at 37°C. 5 μg of sample was incubated with complexes of 10 μl of the S9.6 antibody (Kerafast ENH001) and 30 μl of Dynabeads Protein A for 2 h at 4°C. 1 μg of sample was used as input.

#### Preparation of SKI expression constructs

The *SKIV2L*, *TTC37* and *WDR61* genes were synthesized and cloned into pFastbac1 vectors by GenScript with the addition of 6x histidine tag and 3x flag tag before the *TTC37* gene creating pFB-SKIV2L, pFB-His-Flag-TTC37 and pFB-WDR61 plasmids respectively. All three genes were codon-optimized for expression in insect cells. The pFB-SKIV2L plasmid was further modified by adding a maltose binding protein tag (MBP) before SKIV2L gene making pFB-MBP-SKIV2L plasmid. The plasmids contained a PreScission protease cleavage site in between the tags and the gene. The cloned genes were expressed using bac-to-bac baculovirus expression system in *Spodoptera frugiperda* Sf9 insect cells. Baculoviruses were produced and the cells were infected with optimal ratios of all three viruses and incubated for 52 h. The cells were collected, washed with phosphate buffered saline and frozen in -80°C until purification.

#### Purification of SKI complex

The frozen cell pellet was resuspended in 3 volumes of lysis buffer containing 50 mM Hepes-KOH pH 7.6, 1 mM DTT, 1 mM EDTA, 1:400 protease inhibitor cocktail (P8340, Sigma-Aldrich), 1 mM PMSF and 30 μg/ml Leupeptine for 20 mins at 4°C with gentle agitation. Then, glycerol (final concentration 16% v/v) and NaCl (final concentration 325 mM) were added and the suspension was stirred slowly for further 30 min. The suspension was centrifuged at 48,000g for 30 min to obtain the soluble extract. Next, the clarified extract was incubated with pre-equilibrated amylose resin (NEB) for 60 mins. The protein bound amylose resin was washed with MBP wash buffer containing 50 mM Hepes-KOH pH 7.6, 2 mM Beta-mercaptoethanol, 250 mM NaCl, 10 % v/v glycerol, 1 mM PMSF and 10 μg/ml Leupeptine followed by elution with MBP wash buffer supplemented with 10 mM Maltose. The MBP eluate was then incubated with pre-equilibrated nickel-nitriloacetic acid resin (Ni-NTA agarose, Qiagen) for 60 mins in the presence of 20 mM imidazole. The Ni-NTA resin was washed with Ni-NTA wash buffer containing 50 mM Hepes-KOH pH 7.6, 2 mM Beta-mercaptoethanol, 150 mM NaCl, 10 % v/v glycerol, 0.5 mM PMSF, 40 mM imidazole and eluted with Ni-NTA wash buffer containing 400 mM imidazole. The Ni-NTA eluate was then incubated overnight with PreScission protease to cleave the tags. The cleaved eluate was concentrated using a centrifugal filter (50 kDa cut-off, Amicon) and then loaded onto a Superose 6 Increase 10/300 GL column (GE Healthcare). The peak fraction containing recombinant SKI complex was collected, sub-aliquoted, snap frozen and stored at -80°C until use.

#### Nucleic acid binding assay

Electrophoretic mobility gel shift assay was carried out in a total reaction of 15 μl in a buffer containing 25 mM Hepes-KOH pH 7.6, 2 mM magnesium acetate, 50 mM NaCl, 1 mM DTT, 0.1 mg/ml BSA, 1 nM DNA/RNA substrate (in molecules) and indicated amounts of SKI protein complex. The reactions were assembled on ice, incubated at 37°C for 30 mins and products were separated on a 0.7% w/v agarose gel at 100 V for 60 mins at 4°C. The gel was dried on Hybond-XL paper (GE Healthcare) in gel dryer (Biorad), exposed to phosphor screen (GE Healthcare) and scanned on FLA-5000 (Fujifilm). The gels were quantitated using Imagequant software (Cytiva) and fraction of protein bound DNA for each substrate was plotted.

#### Preparation of DNA/RNA substrates

The sequences of all oligonucleotides and substrates used are listed in Tables S1 and S2. The RNA and DNA oligonucleotides were synthesized and PAGE purified by SIGMA. The oligonucleotides were labelled with radioactive ^32^P at 5’ end by T4 polynucleotide kinase. The labelled oligo was annealed to the complementary non-labelled oligo in a 1:2 ratio in polynucleotide kinase buffer, heated at 95°C for 5 min followed by cooling overnight.

#### Structural analysis of SKIV2L

The structure of human SKIV2L (SKI2W) was extracted from the RNA-bound human SKI complex in the closed state (PDB: 7QDY^38^) and used as the input for structural mutant analysis using the Missense3D mutant prediction server.^43^ The structural changes due to the introduction of the V314G mutation was determined using both the predication results and manual visual inspection of the region. The figures featuring structural data were generated using Open-Source PyMOL^77^ and ChimeraX.^72^

#### FACS analysis

HeLa1.3 cells were incubated with 10 μM of EdU (Thermo Fisher, E10415) for 1 h prior to collection. Then, cell suspensions were fixed with formaldehyde 4% (w/v) in PBS for 15 min with vigorous vortexing to dissociate any remaining cellular clumps; for the same purpose the cells were passed through a syringe with a 0.5 mm needle tip prior to fixation. The Click-iT EdU Alexa Fluor 647 Flow Cytometry Assay kit (Thermo Fisher Scientific, C10634) was then used according to the manufacturer’s instructions. We counterstained DNA with 10 μg/ml of Propidium Iodide (PI) (Sigma-Aldrich, P4864). Cell preparations were analysed with a BD LSR II flow cytometer. HEK293 cells were incubated with 10 μM of EdU for 30 min prior to collection. Cell suspensions were washed with cold PBS, fixed with cold ethanol 70% and stored overnight at 4°C. Then, cells were permeabilised with 0.05% triton in PBS for 10 min at 4°C. The Click-iT EdU Alexa Fluor 488 Imaging kit (Thermo Fisher Scientific, C10337) was then used according to the manufacturer’s instructions. We counterstained DNA with 10 μg/ml of 7-Amino-Actinomycin D (7-AAD) (BD Biosciences, 559925) and treated samples with 0.02 μg/μl RNase A. Cell preparations were analysed with a BD LSRFortessa X-20 flow cytometer.

#### Cell cycle study

Cells were seeded at a 40% confluence in 4-well slides. After the appropriate synchronisation protocol, cells were incubated for 1 h with 10 μM of EdU before collection. No pre-extraction was performed. Furthermore, for collection of mitotic enriched populations, cells were fixed with 2X fixative solution in media for 15 min to prevent excessive detachment of cells. Afterwards, the Click-iT Edu Alexa Fluor 647 Imaging Kit (Thermo Fisher, C10640) was employed to label DNA synthesis following the manufacturer’s indications. Then, a regular immunofluorescence using a primary mouse anti-Cyclin A antibody (1/50, Santa Cruz biotechnology, sc-53227) was performed.

#### Western blotting (WB)

Cells were trypsinised, washed in PBS 1X and spun down at 300 g for 5 min. Cell pellets were subjected to lysis by incubating in lysis buffer (NaCl 40 mM, Tris 25 mM, pH 8; MgCl_2_ 2 mM; SDS 0.05% (w/v), Benzonase 100 units/ml (Sigma-Aldrich, E1014-25KU) and cOmplete, EDTA-free Protease Inhibitor Cocktail 2X) for 10 min on ice. The lysates were sheared by being forced through a 25G needle for 10 times and then incubated on ice for 10 min. Protein concentration was determined using the Bio-Rad Protein Assay Dye Reagent Concentrate (Bio-Rad, 500-0006) according to the manufacturer’s instructions. Protein lysates were denatured for 5 min at 100°C after addition of Laemmli buffer 4X (50 mM Tris pH 6.8; 100 mM DTT; 2% (w/v) SDS; 0.1% (w/v) bromophenol blue; 10% glycerol (v/v)), separated on NuPAGE 4-12% (w/v) Bis-Tris gels and transferred onto a nitrocellulose membrane (Amersham Protran 0.2 μm, GE10600001). The membrane was blocked using non-fat milk 5% (w/v) in PBS-T (PBS 1X; 0.1% (v/v) Tween-20) and subsequently incubated overnight at 4°C with the primary antibody (anti-RNAseH1 (1/500, Abnova, H00246243-M01), anti-GFP (1/5000, Abcam, ab290), anti-β-actin (1/5000, Abcam, ab8226, used as a loading control), anti-H3 (1/1000, Abcam, ab10799), anti-SKIV2L (1/1000, Proteintech group, 11462-1-AP), anti-TTC37 (1/500, Novus Biologicals, NBP1-93640), anti-WDR61 (1/500, Sigma-Aldrich, SAB1401852; 1/1000, Thermofisher PA5-40079), anti-α-Tubulin (1/1000, Sigma-Aldrich, T6199), anti-TRF2 (1/2000, Novus Biologicals, NB110-57130), anti-TRF1 (1/1000, SantaCruz, sc-56807), anti-TIN2 (1/1000, Sigma-Aldrich, SAB4200108)) diluted in non-fat milk or BSA 5% (w/v) in PBS-T. Subsequently, the membrane was washed 3 x 10 min in PBS-T. Following incubations with HRP-conjugated secondary antibody (anti-mouse and anti-rabbit, Agilent, P0447 and P0217) in non-fat milk 5% (w/v) in PBS-T and 3 x 10 min washes in PBS-T, the signal was visualized using ECL Western blotting reagents (Sigma-Aldrich, RPN2106) and either X-ray film exposure (Amersham Hyperfilm ECL Sigma-Aldrich, GE28-9068-35) or Amersham Imager 680 (GE Healthcare).

#### PICh and mass spectrometry analysis

PICh experiments were conducted as previously described^27^^,^^28^ with minor modifications on the sample processing. Protein samples were processed using the Filter Aided Sample Preparation (FASP) protocol.^78^ Briefly, samples were loaded onto 30 kDa centrifugal concentrators (Millipore, MRCF0R030) and buffer exchange was carried out by centrifugation on a bench top centrifuge (15min, 12,000g). Multiple buffer exchanges were performed sequentially with UA buffer (8M urea in 100mM Tris pH 8.5, 3x200 μl), reduction with 10mM DTT in UA buffer (30min, 40°C) and alkylation with 50mM chloroacetamide in UA buffer (20min, 25°C). This was followed by buffer exchange into UA buffer (3x100 μl) and 50mM ammonium bicarbonate (3x100 μl). Digestion was carried out with mass spectrometry grade trypsin (Promega, V5280) using 1 μg protease per digest (18h, 37°C). Tryptic peptides were collected by centrifugation into a fresh collection tube (10 min, 12,000g) and washing of the concentrator with 0.5M sodium chloride (50 μl, 10 min, 12,000g) for maximal recovery. Following acidification with 1% (v/v) trifluoroacetic acid (TFA) to a final concentration of 0.2% (v/v), collected protein digests were desalted using Glygen C18 spin tips (Glygen Corp, TT2C18.96) and peptides eluted with 60% (v/v) acetonitrile, 0.1% (v/v) formic acid (FA). Eluents were then dried using vacuum centrifugation. Raw data available via ProteomeXchange with identifier PXD046955.

#### Liquid chromatography-tandem mass spectrometry

Dried tryptic digests were redissolved in 0.1% TFA by shaking (1200rpm) for 30 min and sonication on an ultrasonic water bath for 10 min, followed by centrifugation (13,000 rpm, 5°C) for 10 min. Liquid chromatography-tandem mass spectrometry (LC-MS/MS) analysis was carried out in technical duplicates and separation was performed using an Ultimate 3000 RSLC nano liquid chromatography system (Thermo Scientific) coupled to a Q-Exactive mass spectrometer (Thermo Scientific) via an EASY spray source (Thermo Scientific). For LC-MS/MS analysis re-dissolved protein digests were injected and loaded onto a trap column (Acclaim PepMap 100 C18, 100μm x 2cm) for desalting and concentration at 8μL/min in 2% acetonitrile, 0.1% TFA. Peptides were then eluted on-line to an analytical column (Acclaim Pepmap RSLC C18, 75 μm × 50 cm) at a flow rate of 250nL/min. Peptides were separated using a 120-minute gradient, 4-25% of buffer B for 90 min followed by 25-45% buffer B for another 30 min (composition of buffer B – 80% acetonitrile, 0.1% FA) and subsequent column conditioning and equilibration. Eluted peptides were analysed by the mass spectrometer operating in positive polarity using a data-dependent acquisition mode. Ions for fragmentation were determined from an initial MS1 survey scan at 70,000 resolution, followed by HCD (Higher Energy Collision Induced Dissociation) of the top 12 most abundant ions at 17,500 resolution. MS1 and MS2 scan AGC targets were set to 3e6 and 5e4 for maximum injection times of 50 ms and 50 ms respectively. A survey scan m/z range of 400 – 1800 was used, normalised collision energy set to 27%, charge exclusion enabled with unassigned and +1 charge states rejected and a minimal AGC target of 1e3.

#### Mass spectrometry raw data processing

Data was processed using the MaxQuant software platform (v1.5.8.3), with database searches carried out by the in-built Andromeda search engine against the Swissprot *H. sapiens* database (version 20170202, number of entries: 20,183). A reverse decoy search approach was used at a 1% false discovery rate (FDR) for both peptide spectrum matches and protein groups. Search parameters included: maximum missed cleavages set to 2, fixed modification of cysteine carbamidomethylation and variable modifications of methionine oxidation, protein N-terminal acetylation, asparagine deamidation as well as glutamine to pyro-glutamate. Label-free quantification was enabled with an LFQ minimum ratio count of 2. ‘Match between runs’ function was used with match and alignment time limits of 1 and 20 min respectively.

#### Northern blotting

RNA extraction was carried out using RNeasy Mini Kit (Qiagen, 74104) and DNA contaminants were eliminated by in column treatment with DNase I (Qiagen, 79254) according to the manufacturer instructions. RNA (20 μg) was denatured for 10 min at 65°C in sample buffer (formamide 50% (v/v), 2.2 M formaldehyde, 1X MOPS) followed by incubation on ice for 5 min. 10X Dye buffer (glycerol 50% (v/v), Bromophenol Blue 0.3% (w/v), 4 mg/ml ethidium bromide) was added to each sample and they were run on a formaldehyde agarose gel (agarose 0.8% (w/v), formaldehyde 6.5% (v/v) in 1X MOPS) at 5 V/cm in 1X MOPS buffer (0.2M MOPS, 50 mM NaOAc, 10 mM EDTA, RNase-free water). The gel was rinsed twice in water, washed 5 min with denaturation solution (1.5 M NaCl, 0.05 M NaOH), followed by three more washes with 20X SSC before transferring the RNA on an Amersham Hybond N+ membrane (GE Healthcare, RPN303B) overnight using a neutral transfer in 20X SSC. The membrane was UV-crosslinked (Stratalinker, 200 J/cm^2^ UV) and baked for 45 min at 80°C. For RNA detection, the membrane was hybridised with a ^32^-P labelled TAA(CCCTAA)_4_ probe using UltraHyb-Oligo solution (Thermo Fisher Scientific #AM8663) at 42°C overnight, followed by 10 min wash with 2XSSC/0.1% (w/v) SDS, 2 min wash with 0.2XSSC/0.1% (w/v) SDS at 42°C and a final 5 min wash with 2XSSC/0.1% (w/v) SDS solution. Radioactive signals were detected by phosphor-imager (Amersham Biosciences).

#### TERRA RT-qPCR analysis

TERRA reverse transcribe (RT)-quantitative (q)PCR analysis was performed as previously described^70^ with some modifications. RNA was extracted as described for Northern blot but two additional DNase I digestions were performed using 1U DNase I (Roche) per μg RNA for 20 min at 37C followed by an in-column treatment with DNase I (Qiagen). RNA was frozen and stored at -80°C. 3 μg of total RNA were used for reverse transcription reactions using 200 U of SuperScript III Reverse Transcriptase (Invitrogen) in a final volume of 20 μl at 55°C for 1 h and enzyme was inactivated at 70°C for 15 min. cDNA was diluted to a final volume of 40 μl and stored at -20°C. TERRAs were reversed transcribed using TERRA specific oligonucleotides (CCCTAACCCTAACCCTAACCCTAACCCTAA). β-Actin gene (*ACTB,* ENSG00000075624) was used as a reference for normalisation. As previously described, random hexamers (Invitrogen, N8080127) were used for reverse transcription.^79^ qPCR reactions (2 μl of cDNA, 5 pmol of each primer and Power SYBR Green PCR Master Mix (Applied Biosystems, 4368708)) were performed in a final volume of 20 μl using a Bio-Rad CFX96 system. 40 cycles of 15s of denaturation at 95°C followed by 1 min of annealing and extension at 60°C were used. qPCR primers are listed in Table S3. Amplicon length for *ACTB* gene is 76bp. Subtelomeric specific primers details were previously described.^11^^,^^70^ The 2_t_^-ΔΔC^ method was used for relative TERRA quantification normalised to the β-Actin gene expression and the shCtr.

### Quantification and statistical analysis

Methods and software used for quantification of PICh, immunofluorescence, PLAs and FISH assays are provided in the respective specific related section of method details. The normalisation used for ChIP, DRIP, Northern blot, qPCR and EMSA data is described in the relevant section of the method details and in the figure legends. Statistical analysis was performed using GraphPad Prism. Error bars, statistical methods, number of data analysed (n) and independent replicates are described in figure legends. SEM: standard error of the mean. Statistical differences were determined by an unpaired two-tailed t test or Mann-Whitney U test with *p* values ≤ 0.05. Figures were prepared using Adobe Illustrator.
