# Supplementary material for: Human SKI component SKIV2L regulates telomeric DNA-RNA hybrids and prevents telomere fragility
Source: iScience. 2024 Oct 4;27(11):111096. doi: 10.1016/j.isci.2024.111096 (PMC11530851; doi:10.1016/j.isci.2024.111096)
Supplement: Document S1. Figures S1–S5 and Tables S1–S3 [file mmc1.pdf]

iScience, Volume *27*

Supplemental information

Human SKI component SKIV2L regulates telomeric DNA-RNA hybrids and prevents telomere fragility

Emilia Herrera-Moyano, Rosa Maria Porreca, Lepakshi Ranjha, Eleni Skourti, Roser Gonzalez-Franco, Emmanouil Stylianakis, Ying Sun, Ruihan Li, Almutasem Saleh, Alex Montoya, Holger Kramer, and Jean-Baptiste Vannier

Herrera-Moyano_FigS1

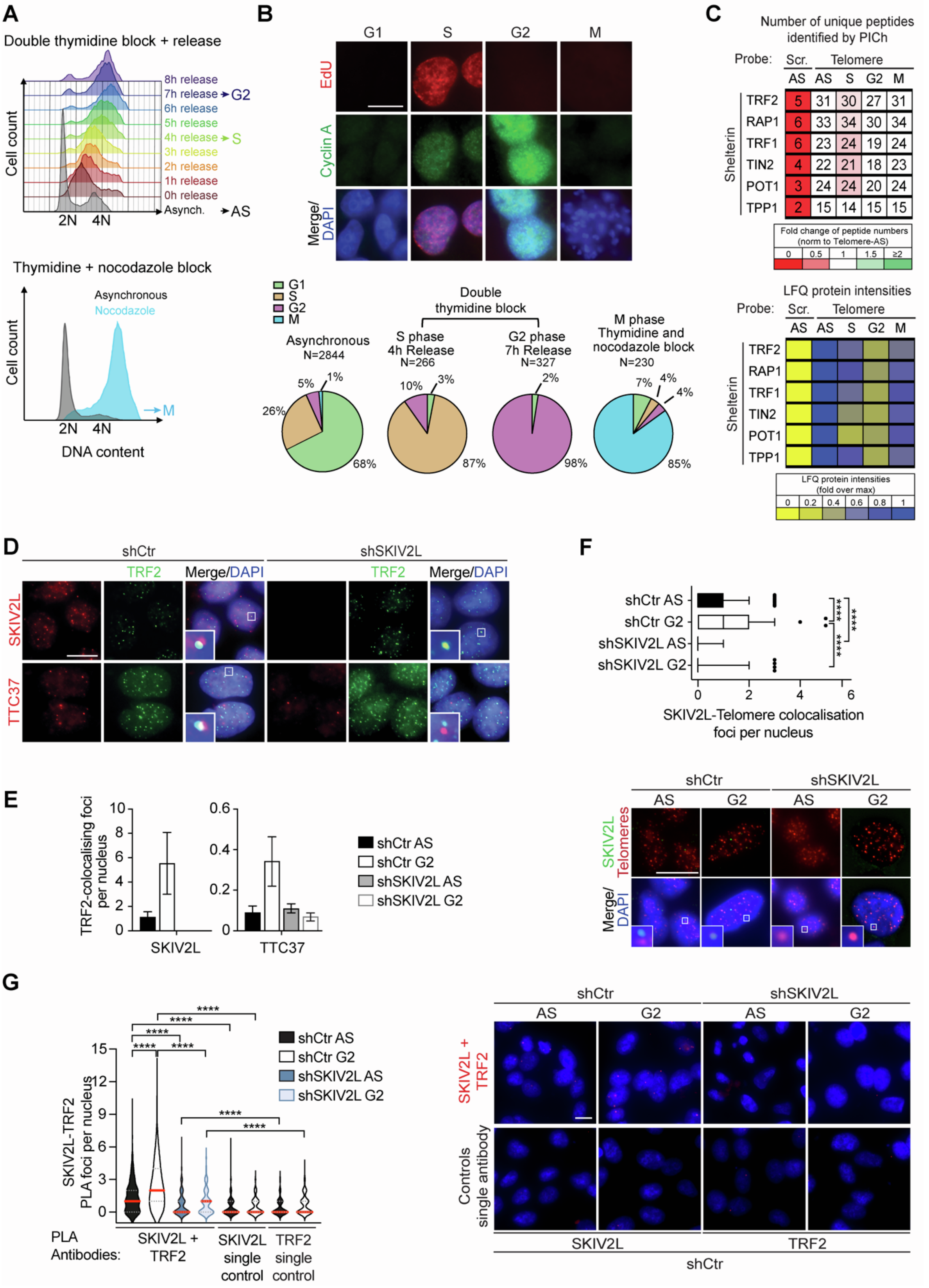

Figure S1. Synchronisation of HeLa1.3 cells and recruitment of SKIV2L to telomeres at different stages of the cell cycle, related to Figure 1. (A) FACS analysis of asynchronous (AS, black) or synchronised (coloured) HeLa1.3 cells using double thymidine block. Cells were released from the

block and collected every hour (for 8 h) for FACS as specified (top panel). FACS analysis of asynchronous (black) and nocodazole blocked (M, blue) HeLa1.3 cells after an 8 h release from thymidine arrest (bottom panel). (B) Representative images of Cyclin A immunofluorescence (IF) (S-G2 phases) and EdU click-it reaction (S phase) in asynchronous HeLa1.3 cells upon incubation with EdU for 1h (top panel). Discrimination between G1 and M phases was based on DAPI signal. Scale bar, 15 μm. Quantification of IF images from AS, synchronised in S phase (4 h release), in G2 (7 h release) and in prometaphase (after 16 h nocodazole block) HeLa1.3 cells (bottom panel). Total number of quantified nuclei per condition (N) and the percentage of cells in each cell cycle phase is indicated. (C) Proteomics of isolated chromatin segments (PICh) analysis showing the binding of Shelterin proteins throughout the cell cycle (AS; S; G2; M) in HeLa1.3 cells. Tables are listing the number of unique peptides isolated, including the fold change values of unique peptides normalised to the asynchronous values (top panel) and the relative LFQ intensity values identified by PICh (bottom panel). Scr: scramble. (D) Representative images of immunofluorescence of pre-extracted cells showing co-localisation of SKIV2L and TTC37 with TRF2 (telomeres) in asynchronous control (shCtr) and SKIV2L-depleted (shSKIV2L) HeLa1.3 cells. Details of colocalising or non-colocalising signals are outlined. (E) Quantification of the numbers of co-localisation foci of SKIV2L and TTC37 with TRF2 per nucleus in asynchronous (AS) and G2-synchronised HeLa1.3 cells (means ± SEM, n = 2 independent experiments). (F) IF-FISH showing co-localisation of SKIV2L and telomeres in asynchronous and G2-synchronised control (shCtr) and SKIV2L-depleted (shSKIV2L) cells (median, Q1 and Q3, n > 210 cells, scale bar 15 μm). Mann-Whitney U test ****p <0.0001. (G) Controls for Proximity Ligation Assay (PLA) experiment of SKIV2L-TRF2 in Figure 1E. Negative controls of PLA foci number per nucleus in shSKIV2L cells and PLA reactions using only one of the two antibodies are depicted (median, Q1 and Q3, 707 (shCtr AS), 638 (shCtr G2), 246 (shSKIV2L AS), 221 (shSKIV2L G2), 457 (shCtr AS only SKIV2L), 406 (shCtr G2 only SKIV2L), 419 (shCtr AS only TRF2), 413 (shCtr G2 only TRF2) cells scored per condition, 4 independent experiments, scale bar 10 μm). Mann-Whitney U test ****p <0.0001.

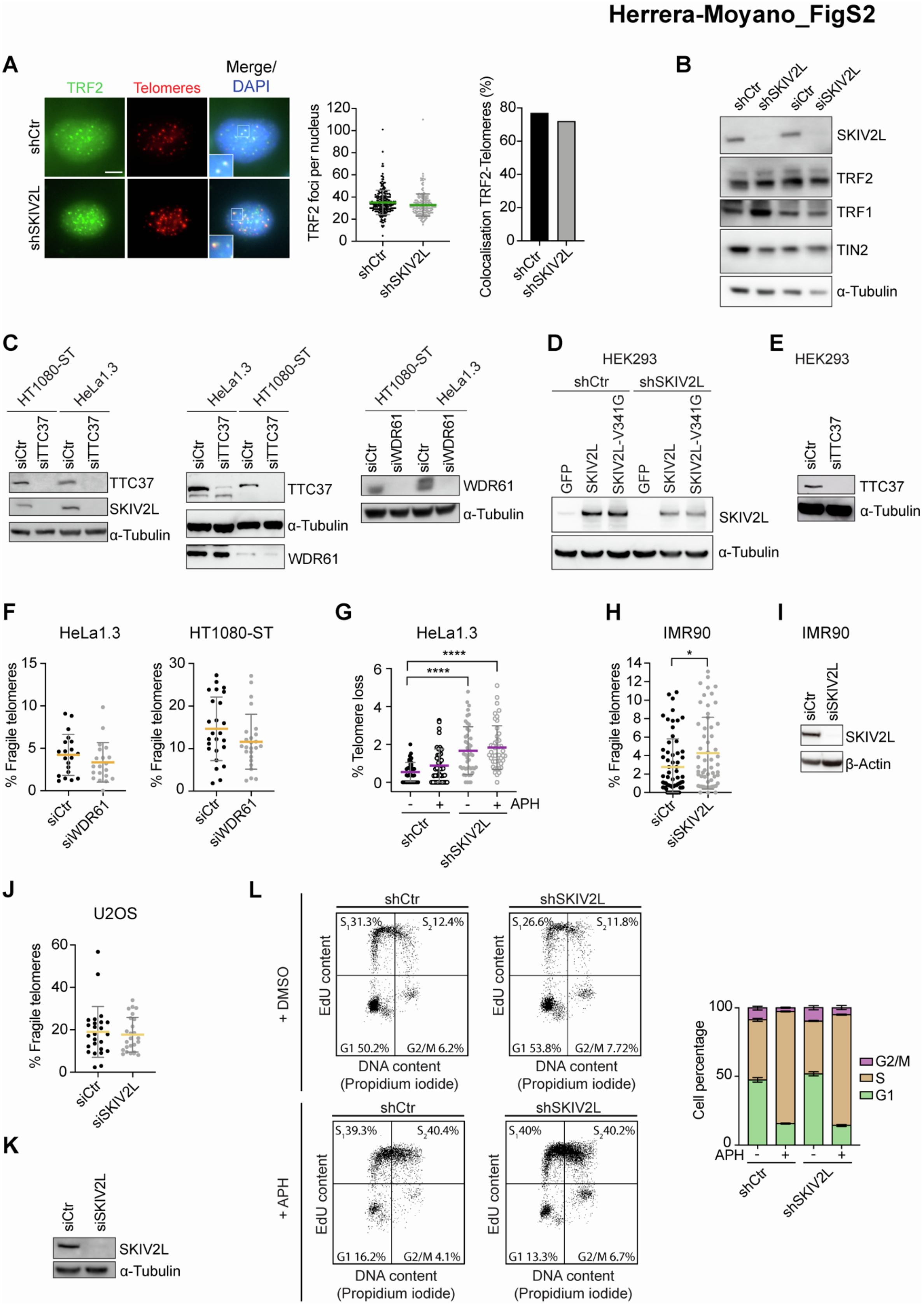

Figure S2. Depletion of SKIV2L leads to telomere fragility but does not perturb Shelterin protein levels, TRF2 binding to telomeres nor cell cycle, related to Figures 1 and 2. (A) IF-FISH showing number of TRF2 foci per nucleus (mean ± SD) and % of TRF2-telomeres co-localisation in control (shCtr) or SKIV2L-depleted (shSKIV2L) HeLa1.3 cells (n = 200 cells, scale bar 5 µm). (B) Western blot

(WB) showing protein levels of SKIV2L, TRF2, TRF1 and TIN2 in control HeLa1.3 cells (shCtr/siCtr) or depleted for SKIV2L (shSKIV2L/siSKIV2L). α-Tubulin was used as a loading control. (C) WB showing protein levels of TTC37, SKIV2L and WDR61 in HeLa1.3 and HT1080-ST cells upon deletion of TTC37 or WDR61. α-Tubulin was used as a loading control. (D) WB showing protein levels of SKIV2L in shCtr and shSKIV2L HEK293 cells overexpressing GFP, a shRNA resistant version of SKIV2L or SKIV2L-V341G. α-Tubulin was used as a loading control. (E) WB showing protein levels of TTC37 in HEK293 cells upon deletion of TTC37. α-Tubulin was used as a loading control. (F) Telomere FISH analysis in WDR61-depleted HeLa1.3 and HT1080-ST cells using siCtr or siWDR61: % of telomere fragility (yellow) per metaphase are represented (means ± SD, n = 20-25 metaphases). (G) Telomere FISH analysis in HeLa1.3 cells using shRNAs: % of telomere loss (purple) per metaphase in shCtr and shSKIV2L treated with DMSO (-, control) or APH (+) (n > 45 metaphases, 2 independent experiments). t test, **** p <0.0001. (H) Telomere FISH analysis in SKIV2L-depleted IMR90 cells: % of telomere fragility (yellow) per metaphase in siCtr and siSKIV2L (means ± SD, n > 57 metaphases, 2 independent experiments). t test *p<0.05. (I) WB showing protein levels of SKIK2L in IMR90 cells upon deletion of SKIV2L. β-Actin was used as a loading control. (J) Telomere FISH analysis in SKIV2L-depleted U2OS cells: % of telomere fragility (yellow) per metaphase in siCtr and siSKIV2L (means ± SD, n = 25 metaphases) (K) WB showing protein levels of SKIK2L in U2OS cells upon deletion of SKIV2L. α-Tubulin was used as a loading control. (L) Flow cytometry analysis of the cell cycle distribution in shCtr and shSKIV2L HeLa1.3 cells untreated (DMSO) or APH treated. DNA was stained with propidium iodide and cells were incubated with EdU to mark newly synthesised DNA. The percentage of cells in G1, early S phase (S_1), late S phase (S_2) and G2/M phases is indicated (mean ± SEM, n=10000 cells, 3 independent experiments, data from S1 and S2 are merged as dataset S).

Herrera-Moyano_FigS3

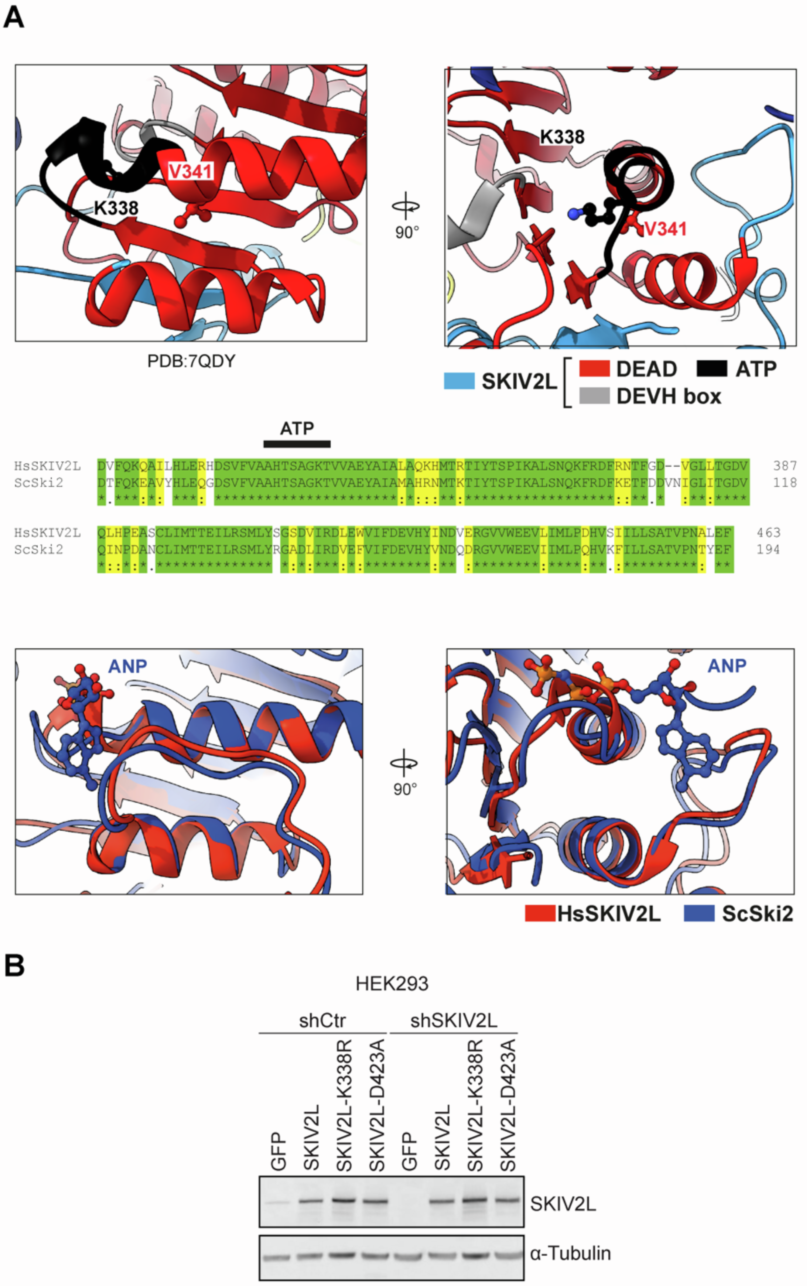

Figure S3. SKIV2L helicase activity is dispensable for its telomeric function, related to Figure 3. (A) Zoom into the ATP binding motif region of SKIV2L. The lysine residue (K338) is localised within the ATP binding motif and is essential for ATP binding. Neighboring valine residue (V341) that is substituted to a glycine residue in a mutant of interest. The DEAD/DEAH box helicase (DEAH) domain and ATP binding (ATP) domain regions are colored according to the key shown. Sequence alignment, using Clustal Omega, of the DEAD domain (harboring an ATP binding motif) of the human (Hs - *H. sapiens*) SKIV2L protein and the yeast (Sc - *S. cerevisiae*) homolog Ski2. Highly conserved residues are coloured with a vertical green line and indicated by the symbol (*), highly similar residues are coloured with a vertical yellow line and indicated by the symbol (:), and residues with similar properties are indicated by the symbol (.). Overlay of human SKIV2L DEAD domain structure and yeast Ski2 DEAD domain structure bound to ANP. (B) WB showing protein levels of SKIV2L in shCtr and shSKIV2L HEK293 cells overexpressing GFP, a shRNA resistant version of SKIV2L, SKIV2L-K338R or -D423R. α-Tubulin was used as a loading control.

Herrera-Moyano_FigS4

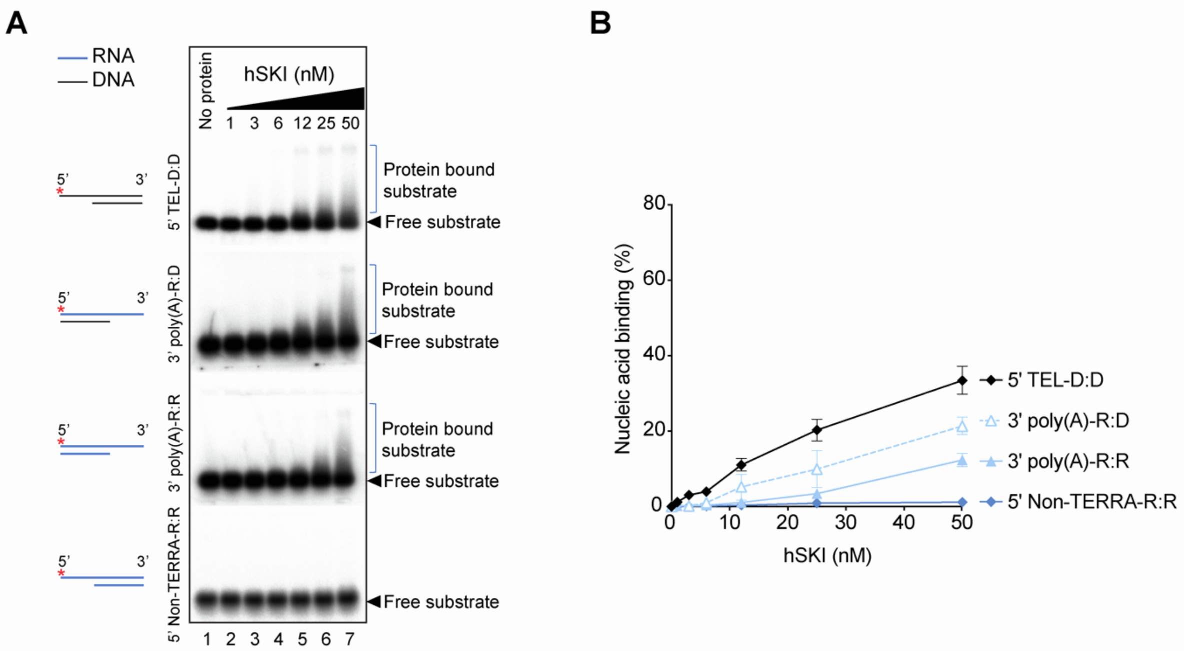

Figure S4. Purified hSKI complex exhibits low binding affinity for non-telomeric 3' overhang substrates and 5' overhang substrates, related to Figure 4. (A) Electrophoretic mobility shift assays showing binding of hSKI complex to different 3' and 5' overhang RNA/DNA substrates. Blue lines denote RNA, black lines denote DNA and asterisk indicates radioactive ^{32}P label. hSKI protein amounts are as indicated (nM). (B) Quantification of (A) showing % of nucleic acid binding calculated as protein bound substrate signal relative to free substrate signal (mean ± SEM, n = 2).

Herrera-Moyano_FigS5

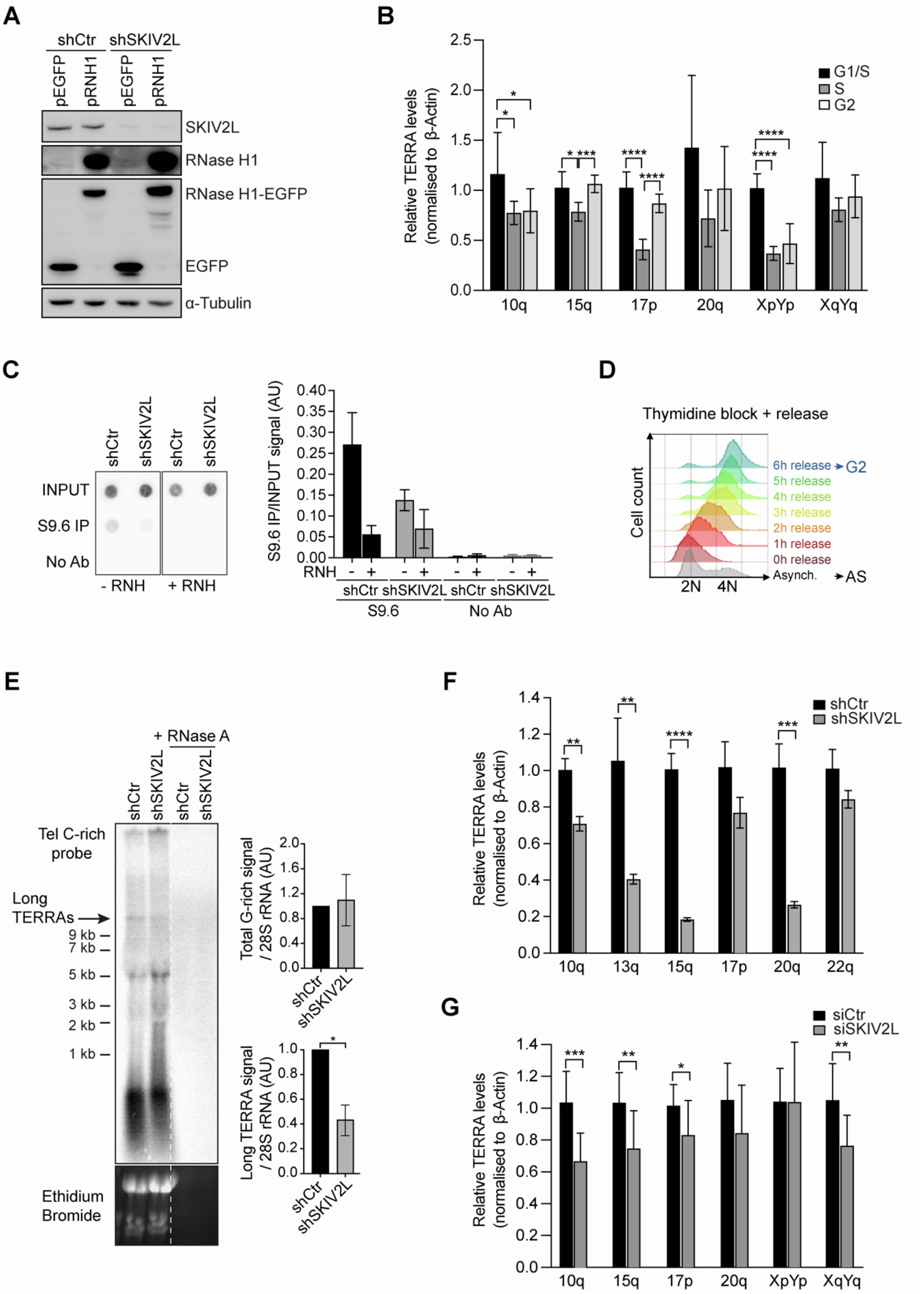

Figure S5. SKIV2L regulates telomeric DNA-RNA hybrids, related to Figure 5. (A) WB analysis showing the levels of RNase H1-EGFP (using anti-GFP and anti-RNase H1 antibodies), EGFP and SKIV2L upon transfection of shCtr and shSKIV2L HeLa1.3 cells with the pRNH1 or pEGFP constructs.

α-Tubulin was used as a loading control. (B) RT-qPCR analysis showing relative TERRA levels from 10q, 15q, 17p, 20q, XpYp and XqYq chromosome ends in G1/S boundary (double-thymidine synchronised cells), S (4 hours release) and G2 (7 hours release) HeLa1.3 cells (means ± SD, n = 4-9, 3 independent experiments). t test * p<0.05, ***p <0.001, ****p <0.0001. (C) DRIP showing the amount of DNA-RNA hybrids at telomeres in asynchronous HeLa1.3 cells using sonicated nucleic acids. RNH, RNase H treatment (means ± SEM, n = 4 independent experiments). Immunoprecipitations without using antibodies were using as a negative control (n = 2). (D) FACS analysis of asynchronous (AS, black) or synchronised (coloured) HEK293 cells using thymidine block. Cells were released from the block and collected every hour (for 6 h) for FACS as specified. (E) TERRA detection by Northern blotting in shCtr and shSKIV2L HeLa1.3 cells. ^{32}P-labelled Tel C-rich probe was used to detect TERRA. Ethidium bromide staining of the 28S rRNA was used as a loading control. Arrow is pointing to the long TERRA band. Half of each sample was treated with Ribonuclease A (+ RNase A) to control for DNA contamination. Total TERRA signals (up) or long TERRA RNA signals were normalised to 28S rRNA signals, relative to shCtr untreated (mean ± SEM, n = 3 independent experiments). (F) RT-qPCR analysis showing relative TERRA levels from 10q, 13q, 15q, 17p, 20q and 22q chromosome ends in control (shCtr) and stable SKIV2L knockdown (shSKIV2L) HeLa1.3 cells (means ± SD, n = 3). t test ** p<0.01, ***p <0.001, ****p <0.0001. (G) RT-qPCR analysis showing relative TERRA levels from 10q, 15q, 17p, 20q XpYp and XqYq chromosome ends in control (siCtr) and transiently SKIV2L-depleted (siSKIV2L) HeLa1.3 cells (means ± SD, n = 3). t test * p<0.05, ** p<0.01, ***p <0.001.

Table S1. DNA/RNA substrates used in this study, related to Figure 4.

	Duplex substrates	Oligonucleotides used
1	dsRNA	ss poly(A) + Css27
2	dsDNA	TEL30 + CTEL30
3	3’ TEL- D:D	cLR01_20 + LR01
4	3’ TERRA- R:R	cLR01_20 RNA + LR01 RNA
5	3’ TERRA- R:D	cLR01_20 + LR01 RNA
6	3’ TEL- D:R	cLR01_20 RNA + LR01
7	3’ Non-TERRA- R:D	5’ss27 + Css17 DNA
8	3’ poly(A)- R:D	ss poly(A) + Css17 DNA
9	3’ poly(A)- R:R	ss poly(A) + Css17
10	3’ Non-TERRA- R:R	5’ss27 + Css17
11	5’ TEL- D:D	TEL30 + 5’CTEL20
12	5’ Non-TERRA- R:R	5’ss27 + 5’Css17

Table S2. Oligonucleotides used in this study, related to Figure 4.

		Name	Sequence 5’ to 3’
1	DNA	TEL18	GGTTAGGGTTAGGGTTAG
2	DNA	Non-TEL18	AGCGTATCCGTTCAGTTG
3	DNA	TEL30	GGTTAGGGTTAGGGTTAGGGTTAGGGTTAG
4	DNA	CTEL30	CTAACCCTAACCCTAACCCTAACCCTAACC
5	DNA	5’CTEL20	CTAACCCTAACCCTAACCCT
6	DNA	ss poly(A) DNA	CCCCACCACCATCACTTAAAAAAAAAA
7	DNA	Css17 DNA	AAGTGATGGTGGTGGGG
8	DNA	LR01	ACGTCAGATGATCGATGGGGTTAGGGTTAG
9	DNA	cLR01_20	CCCCATCGATCATCTGACGT
10	RNA	ss poly(A)	CCCCACCACCAUCACUUAAAAAAAAAA
11	RNA	Css27	UUUUUUUUUUAAGUGAUGGUGGUGGGG
12	RNA	Css17	AAGUGAUGGUGGUGGGG
13	RNA	5’ss27	CCCCACCACCAUCACUUACCAUCACUU
14	RNA	5’Css17	AAGUGAUGGUAAGUGAU
15	RNA	TERRA	UUAGGGUUAGGGUUAGGGUUAGGGUUAGGG
16	RNA	LR01 RNA	ACGUCAGAUGAUCGAUGGGGUUAGGGUUG
17	RNA	cLR01_20 RNA	CCCCAUCGAUCAUCUGACGU

Table S3. qPCR primers used in this study, related to Figure 5.

	Name	Sequence 5' to 3'
1	10q_Forward	AACCTGAACCCTAACCCTCC
2	10q_Reverse	ATTGCAGGGTTCAAGTGCAG
3	13q_Forward	CTGCCTGCCTTTGGGATAA
4	13q_Reverse	AAACCGTTCTAACTGGTCTCTG
5	15q_Forward	CAGCGAGATTCTCCCAAGCTAAG
6	15q_Reverse	AACCCTAACCACATGAGCAACG
7	17p_Forward	GATCCCACTGTTTTTATTACTGTTCCT
8	17p_Reverse	GGGACAGAAGTGGATAAGCTGATC
9	20q_Forward	GCAGCTTTCTCAGCACAC
10	20q_Reverse	TTTGTTCACTGTCGATGCG
11	22q_Forward	CGAAACAGAACCCGAAGCAG
12	22q_Reverse	TGCACACATGACACCCAAAA
13	XpYp_Forward	GCAAAGAGTGAAAGAACGAAGCTT
14	XpYp_Reverse	CCCTCTGAAAGTGGACCAATCA
15	XqYq_Forward	TCCTAATGCACACATGATACCC
16	XqYq_Reverse	CCCTAAGCACATGAGGAATGT
17	Actin_Forward	GCTACGAGCTGCCTGACG
18	Actin_Reverse	GGCTGGAAGAGTGCCTCA
